# Feeding regime synchronizes circadian clock in choroid plexus - insight into a complex mechanism

**DOI:** 10.1007/s00018-025-05798-3

**Published:** 2025-06-23

**Authors:** Tereza Dočkal, Pavel Houdek, Kateryna Semenovykh, Revan Rangotis, Martin Sládek, Alena Sumová

**Affiliations:** 1 Laboratory of Biological Rhythms, Institute of Physiology of the Czech Academy of Sciences, Videnska 1083, 14200 Prague, Czech Republic; 2 Faculty of Science, Charles University, Prague, Czech Republic; 3 Second Faculty of Medicine, Charles University, Prague, Czech Republic

**Keywords:** Choroid plexus, Circadian clock, Restricted feeding, Insulin, Glucose, Temperature, O-GlcNAc

## Abstract

The circadian clock in choroid plexus (ChP) controls processes involved in its physiological functions, but the signals that synchronize the clock have been sparsely studied. We found that the ChP clock in the fourthventricle (4V) is more robust than that in the lateral ventricle (LV) and investigated whether both clocks use information about mealtime as a signal to synchronize with the current activity state. Exposure of *mPer2*^*Luc*^ mice to a 10-day reverse restricted feeding (rRF) protocol, in which food was provided for 6 h during daytime, advanced the phase of the ChP clock in 4V and LV, as evidenced by shifted (1) PER2-driven bioluminescence rhythms of ChP explants ex vivo and (2) daily profiles in clock gene expression in both ChP tissues in vivo. In contrast, clocks in other brain regions (DMH, ARC, LHb) of the same mice did not shift. The 4V ChP responded more strongly than the LV ChP to rRF by modulating the expression of genes to ensure a decrease in resistance to cerebrospinal fluid drainage and increase the secretory capacity of ChP cells. Mechanistically, rRF affects the ChP clock through food-induced increases in insulin, glucose and temperature levels, as in vitro all three signals significantly shifted the clocks in both ChP tissues, similar to rRF. The effect of glucose was partially blocked by OSMI-1, suggesting involvement of O-linked N-acetylglucosamine posttranslational modification. We identified mechanisms that can signal to the brain the time of feeding and the associated activity state via resetting of the ChP clock.

**Graphical abstract:**

Summary of results. Reverse restricted feeding (rRF), which shifts the timing of food intake into the daytime, leads to a corresponding shift in the rise of insulin and glucose levels as well as activity-related body temperature. As a result, the clocks in the choroid plexus of the fourth ventricle (4V ChP) and the lateral ventricle (LV ChP) shift accordingly (the effect of glucose is partly mediated via O-GlcNAcylation). In this way, clock-controlled ChP function follows the timing of food rather than solar cycle.

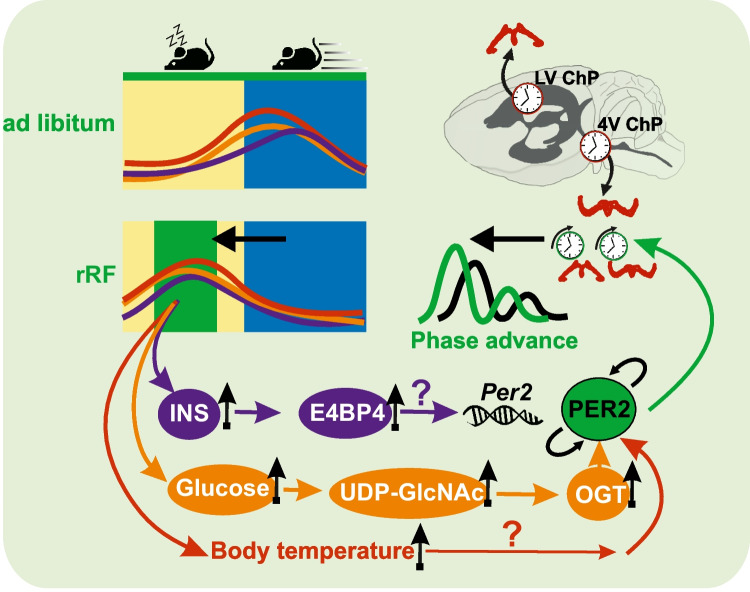

**Supplementary Information:**

The online version contains supplementary material available at 10.1007/s00018-025-05798-3.

## Introduction

Choroid plexus (ChP) is a highly vascularized non-neural tissue in the third, fourth, and lateral brain ventricles. It produces cerebrospinal fluid (CSF) by actively filtering blood plasma through a monolayer of epithelial cells bound by the tight junction [1] and forming a barrier that prevents paracellular diffusion of solutes from the blood into the CSF and vice versa [2, 3]. Because of its unique structural organization, localization, and involvement in various transport and secretory pathways, ChP is the major control unit for the cellular composition of CSF and brain fluids [4]. Similar to other cells and tissues in the body, ChP harbors a robust circadian clock that exhibits rhythmicity both in vivo as well as when explanted into culture in vitro [5–8]. At the cellular level, autonomous circadian rhythmicity is driven by transcriptional-translational feedback loops (TTFL) that control the expression of a set of clock genes (*Per1,2, Cry1,2, Bmal1,2, Clock, Nr1d1,2*) [9]. The resulting rhythmic changes in levels of their protein products provide the link to the temporal control of tissue-specific, physiologically relevant genes [10].

Recently, a role of the circadian clock in regulating ChP-specific functions has been ascertained in a study, which showed that the ChP clock regulates rhythmically many ChP-specific processes, including the CSF circadian secretome, endoplasmic reticulum stress response, and expression of genes involved in neurodegenerative diseases (Alzheimer’s disease, Huntington’s disease, and frontotemporal dementia) [11]. The local ChP clock and the clock-driven rhythms rely on rhythmic signals originating from the central circadian clock located in the suprachiasmatic nuclei (SCN) in the anterior part of the hypothalamus [11]. The mechanisms by which the SCN communicates with the ChP clock may include the SCN-driven rhythm in levels of glucocorticoid hormones, as their synthetic analog dexamethasone can efficiently reset the ChP clock in the adrenalectomized rats [8] and the SCN-lesioned mice [11]. Dexamethasone also significantly increases the amplitude and efficiently resets the phase of the ChP clock [8, 11].

However, other pathways involved in the communication between the rest of the body and the ChP clock remain to be explored. In this study, we tested the hypothesis that the circadian clock in the ChP responds to changes in feeding regime. The hypothesis is based on the assumption that the ChP clock would be sensitive to the changes in the feeding/fasting cycle, as has been shown for other peripheral clocks elsewhere in the body [12]. In the brain, feeding adjusts the phase of various neuronal extra-SCN oscillators, such as in the arcuate nucleus, the paraventricular nucleus [13, 14], the ventromedial nuclei [15] or the dorsal and ventral striatum [16–20]. Feeding during the resting phase leads to internal desynchrony due to uncoupling of the SCN clock from the peripheral clocks [12] with adverse health implications [21]. Considering the significant role of the local ChP clock in multiple ChP functions and its dependence on the signals from the SCN [11], information on whether and how the ChP clock responds to changes in the feeding/fasting cycle is of utmost importance, as it may underlie the effects of misaligned feeding on disruption of brain homeostasis. To date, the sensitivity of the ChP clock to the feeding regime has only been marginally investigated [22], with the mechanism of the effect and ventricle-specific ChP sensitivity to the feeding/fasting regime yet to be determined.

Therefore, to fill the gap, we examined the effects of 10 day-reversed restricted feeding (rRF), during which mice had access to food restricted to only 6 h during the light phase of day (when they normally sleep), on the ChP clock using ex vivo, in vitro and in vivo approaches. We tested a battery of potential mechanisms involved in the effect of feeding on the ChP clock, such as changes in glucose, temperature, selected feeding-related hormones, and osmolarity, of which we identified changes in glucose levels in the CFS as the most potent mediator that may affect the ChP clock via O-GlcNAcylation.

## Materials and methods

### Animals

Adult male *mPer2*^*Luc*^ mice (strain B6.129S6-Per2tm1Jt/J, JAX, USA; a colony held at the Institute of Physiology, the Czech Academy of Sciences) were individually housed under a 12 h light/12 h dark cycle (LD12:12); lights on at 6:00 was assigned as Zeitgeber time (ZT) 0 and lights off at 18:00 was assigned as ZT12. Mice of the control group (Ctrl) had unlimited access to food and water (ad libitum feeding). Mice of the group subjected to reversed restricted feeding (rRF) for 10 days had access to food only for 6 h during the light period of the LD12:12 (ZT3—ZT9).

### Monitoring of body weight and food and water intake

Mice of the Ctrl and rRF groups were weighted before and after the rRF protocol (*n* = 18 per group), and food consumed was weighted every day (*n* = 12 per group) (Supplementary Fig. S2A). After an initial decrease in food intake during the first 4 days, the mice adapted to the situation and consumed the same amount of food as the ad libitum-fed controls during the second half of the protocol. Body weight decreased in the mice exposed to the rRF (paired t-test; *P* = 0.004), but not in the controls (paired t-test; *P* = 0.0673). Mice of both groups (*n* = 3 per group) were monitored continuously with infrared cameras attached above each cage, and the time they spent eating and drinking over 3 days was calculated from the video by an observer blind to the procedure. The mean values of the 24 h profiles for each mouse were calculated (Supplementary Fig. S2B). Data are expressed as mean ± SD of the 3 mice for each group.

### Collection of samples, RNA isolation and real-time RT-qPCR

Mice from the Ctrl and rRF groups were sacrificed at 7 time points (*n* = 5 for each time point) during the 24 h cycle (ZT0, ZT4, ZT8, ZT12, ZT16, ZT20, ZT24). From each brain, one sample of ChP was immediately removed from the lateral ventricle (LV) and one from the fourth ventricle (4V). Subsequently, the brains were frozen and three additional brain regions, namely dorsomedial hypothalamus (DMH), nucleus arcuatus (ARC), and lateral habenula (LHb), were isolated using a laser microdissector (LMD6000, Leica) as described previously [23]. Dissected samples from each brain were collected in a microtube containing RLT buffer from the RNeasy Micro kit (QIAGEN, Valencia, USA) and stored until RNA isolation. Total RNA was isolated using the RNeasy® Micro Kit (QIAGEN) and the final concentration was measured using the NanoDrop One Microvolume UV–Vis Spectrophotometer (Thermo Scientific). Characterization of RNA and measurement of RNA quality and integrity was performed using Agilent Bioanalyzer (Agilent RNA 6000 Pico Kit). RNA was then reverse-transcribed into cDNA using the High-Capacity cDNA Reverse Transcription Kit (Applied Biosystems™) according to the working protocol. The cDNA samples were analyzed by RT-qPCR reaction using the Xceed qPCR SG 2 × Mix Lo-ROX kit (IAB, Prague, Czechia) with predesigned KicqStart primers (Merck; Darmstadt, Germany). Relative expression was quantified using the Livak’s ΔΔCt method against the geometric mean of three reference genes (*B2m, Tbp, Pgk1*) and against one standard sample. For the list of primers, see Supplementary Fig. S1. Data are expressed as the mean ± S.E.M.

### Cerebrospinal fluid collection and glucose level measurement

Mice were terminally anesthetized using thiopental (64 mg/kg) at ZT6 or ZT18. For collection of the CSF samples, the scalp was removed and superficial neck muscles were cut with a scalpel between the scull base and atlas. After muscle removal, the atlanto-occipital membrane above the cisterna magna and dura mater was punctured by a glass capillary to aspire CSF (volume of 10 µl). The blood samples (volume of 30 µl) were obtained from the tail vein. The glucose levels were measured using glucose meter (Glucolab auto-coding glucose meter, MEDITEST, Czech Republic).

### Organotypic explants and bioluminescence recordings ex vivo

Mice were sacrificed at ZT6 – ZT7, brains were sectioned into 300-µm coronal sections using vibratome (Leica, Wetzlar, Germany) and ChP explants from the 4 V and LV were dissected. Explants were immediately placed on Millicell Culture Inserts (Merck; Darmstadt, Germany) in 35-mm petri dishes containing 1 ml of air-buffered recording medium (DMEM supplemented with 100 U/ml penicillin, 100 µg/ml streptomycin, 1% GlutaMAX (all Thermo Fisher; Waltham, MA, USA), 2% B27 supplement (Thermo Fisher) and 0.1 mM D-Luciferin (Biosynth, Staad, Switzerland). Bioluminescence traces were recorded in Lumicycle (Actimetrics; Wilmette, IL, USA) and analyzed using LumiCycle Analysis software (Actimetrics). Data were baseline-subtracted using the 24-h running average and fitted with a damped sine wave to calculate period and phase. The amplitude was calculated for each bioluminescence peak (max - min/2) and referred to as a peak amplitude.

### Treatments and constructing phase response curve

ChP explants prepared as described above were cultured in fresh recording media and bioluminescence was recorded in a Lumicycle for 3 days. On the 4th day, explants were subjected to a treatments at various time points throughout the bioluminescence rhythm. Treatments involved administration of insulin (500 nM), ghrelin (50 ng/ml), leptin (20 nM), sodium chloride (5.3 mM), sodium gluconate (0.5%), OSMI-1 (100 µM) or vehicle (H_2_O or DMSO). For in vitro testing of sensitivity to glucose, we cultivated explants with different glucose concentration (0, 3.5, 12.5, or 25 mM), representing extremely low, physiologically relevant, and high concentration. Relative amplitudes and periods were calculated as the ratio of amplitude and period values detected after and before the treatments. The phase shift caused by the treatments was quantified by fitting a sine curve to at least three complete circadian cycles of a 24-h running average of the baseline-subtracted rhythm before treatment, extrapolating beyond the time of treatment, and calculating the difference between the time of the first peak of the extrapolated rhythm and the actual peak after treatment. For construction of the phase response curves (PRCs), insulin, leptin, ghrelin and vehicle (H_2_O) were applied at different times over 24 h. The phase shifts, referred to as phase advances (+) or phase delays (-), were calculated with normalization to the endogenous period and plotted as a function of treatment time relative to the trough (time 0) or peak (time 12) of the bioluminescence rhythm.

### PER2 degradation assay

ChP explants were cultured in fresh recording media with two different glucose concentrations (3.5 mM, 25 mM), treated with OSMI-1 (100 µM) or vehicle (DMSO) and bioluminescence was recorded in Lumicycle. When the second peak in PER2-driven bioluminescence was reached, inhibitor of translation, cycloheximide (CHX, 40 µg/ml, Merck; Darmstadt, Germany), was added and the bioluminescence was recorded for following 10 h. One-phase exponential decay curves were fitted. Afterwards, the explants were exposed to washing procedure and recording continued in fresh media for the next 10 h. Half-life was quantified by fitting exponential decay curves to the first 10 h of luminescence data post CHX treatment. The rate of PER2 accumulation was expressed as the time necessary to reach the first bioluminescence peak of each individual explants after washing procedure.

### Temperature entrainment

ChP explants were monitored in the Lumicycle kept inside an incubator (Binder, Germany) with programmable temperature; the temperature inside the Lumicycle was recorded in real time with a GMH3210 digital thermometer (Greisinger Electronic, Germany). The temperature protocol (T-protocol) consisted of monitoring the explants at a basal temperature of 37 °C for 3 cycles, followed by 3 cycles with an abrupt (square wave) increase in temperature to 39 °C for 3 h and a decrease back to 37 °C for the rest of the day (21 h) and finally in the initial temperature of 37 °C for 3 cycles. Control explants were maintained at basal temperature (37 °C) throughout the experiment. The effect of T-protocol on the amplitude and period measured before (B) and after (A) the temperature intervention was compared. In a separate experiment, T-protocol was applied at different times over the 24 h cycle and the change in phase of the bioluminescence rhythm was calculated. The PRC was constructed as described above to visualize the dependence of the phase shift on the time of initiation of the T-protocol.

### Statistical analysis

Presence or absence of circadian rhythm in daily profiles of gene expressions was assessed by 1-way ANOVA for the effect of time, and by cosinor analysis by fitting two alternative regression models: horizontal straight line (null hypothesis) or cosine curve defined by the equation Y = mesor + (amplitude × cos(2 × π × (X − acrophase)/wavelength)) with a constant wavelength of 24 h (alternative hypothesis) [24]; P values, R2 (goodness of fit), amplitudes and acrophases were determined. 2-way ANOVA with Sidak’s multiple comparisons was used for the detection of differences in the gene expression profiles between groups. For the bioluminescence rhythms, the relative periods and amplitudes were compared by 1-way ANOVA with Tukey’s multiple comparisons. Circadian parameters (amplitude, period) were measured before and after the treatments and their ratios were evaluated by Wilcoxon matched-pairs signed rank test and Mann–Whitney test for ranks of independent samples, 1-way ANOVA, or 2-way ANOVA for group difference. To create the PRCs, data were binned into 3 h intervals and compared using 2-way ANOVA. All statistics were performed using GraphPadPrism 7 software (GraphPad, CA USA).

## Results

### Clock in the 4V ChP is more robust compared to the clock in the LV ChP under control conditions

**Fig. 1:**
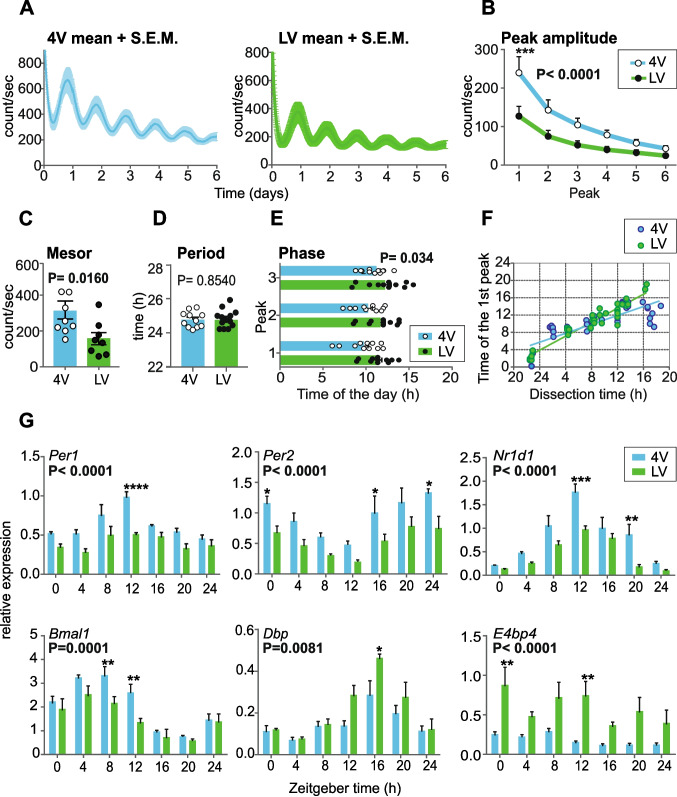
Comparison of circadian parameters of clocks in the ChP of the fourth ventricle (4V) and lateral ventricle (LV). **A** PER2-driven bioluminescence of ChP explants from 4V and LV recorded over 6 days (mean ± S.E.M., *n* = 10) **B** Amplitudes of individual peaks (peak amplitude) of PER2-driven bioluminescence of 4V and LV explants (*n* = 10; 2-way ANOVA; *P* < 0.0001; post hoc ***P = 0.0008). **C** Mean level (mesor) of PER2-driven bioluminescence of 4V and LV explants (*n* = 8; Mann–Whitney test; *P* = 0.0160). **D** Periods of PER2-driven bioluminescence of 4V and LV explants (*n* = 12; Mann–Whitney test; *P* = 0.8540). **E** Phase of PER2-driven bioluminescence of 4V and LV explants expressed as the time of day when the first endogenous peak occurred (*n* = 12; 2-way ANOVA, *P* = 0.0034) is not affected by time of dissection of the explant. **F** Time of the 1 st peak of the PER2-driven bioluminescence rhythms in relation to time of the explant dissection (F-test, *P* = 0.0001, n(4V) = 56, n(LV) = 37). **G** Gene expression levels in 4V and LV ChP tissues collected every 4 h over a 24-h period (*n* = 5 per 1 time point; 2-way ANOVA). All 2-way ANOVAs were performed with Sidak´s multiple comparison; *P < 0.05, **P < 0.01, ***P < 0.001, ****P < 0.0001. All values are means ± S.E.M

First, we compared the circadian characteristics of the clocks in 4V ChP and LV ChP of Ctrl animals fed ad libitum using ex vivo approach by analysing parameters of the PER2-driven bioluminescence rhythms in ChP explants cultured for 6 days (bioluminescence rhythms are shown as mean ± S.E.M. in Fig. 1A, *n*= 10 per each tissue) (Fig. 1A-F). Compared to LV ChP explants, the 4V explants had significantly higher amplitudes of each peak (peak amplitude) (2-way ANOVA, *P* < 0.0001) (Fig. 1B), as well as higher mesors (Mann–Whitney test; *P* = 0.0177) (Fig. 1C). Period did not differ between both ChP explants (Mann–Whitney test; *P* = 0.9851, 4 V: 25.15 ± 0.1476; LV: 25.36 ± 19.34) (Fig. 1D). Additionally, the phases of the first three peaks of the 4V explants were earlier than those of LV explants (2-way ANOVA; *P* = 0.034) (Fig. 1E). Testing the robustness of the clocks in ChP ex vivo [25] showed that the timing of the first peak of PER2 in LV ChP was more dependent on the time of explant preparation than in 4V ChP (F-test, *P* = 0.0001, n(4V) = 56, n(LV) = 37) (Fig. 1F), confirming the higher robustness of the 4V ChP oscillator.

Next, we analysed the clock gene expression profiles in both ChP tissues collected every 4 h over 24 h period. The circadian rhythm was confirmed by significant result of cosinor analysis and 1-way ANOVA (for more details, see Materials and Methods) for *Per2*, *Nr1d1* and *Bmal1* in both ChP tissues, but *Per1* was rhythmically expressed only in 4V ChP (Fig. 1G; Supplementary Fig. S2). Similar to ex vivo data, amplitudes of the rhythms in expression of core clock genes were higher in the 4V ChP than in the LV ChP. Interestingly, amplitude of the rhythmic expression of clock-controlled gene *Dbp* was significantly lower and the *E4bp4* expression (non-rhythmic in LV ChP) was downregulated in the 4V ChP compared to the LV ChP (results of 2-way ANOVA are shown in Fig. 1G; results of cosinor analysis and 1-way ANOVA are shown in Supplementary Fig. S2– ad libitum). In contrast to the ex vivo results (Fig. 1E), phases of the rhythmic gene expression profiles for 4V and LV ChP were similar, likely due to lower temporal resolution of the method (Supplementary Fig. S2– acrophases).

These results indicate significant differences in robustness of the clocks in the 4V and LV, as both ex vivo and in vivo approaches confirmed higher amplitudes of clock rhythmicity in the 4V than in the LV ChP. However, clock in the LV ChP may drive rhythms in expression of output genes with a higher amplitude.

### Exposure of mice to rRF affects the ChP clock in a ventricle-specific manner

**Fig. 2:**
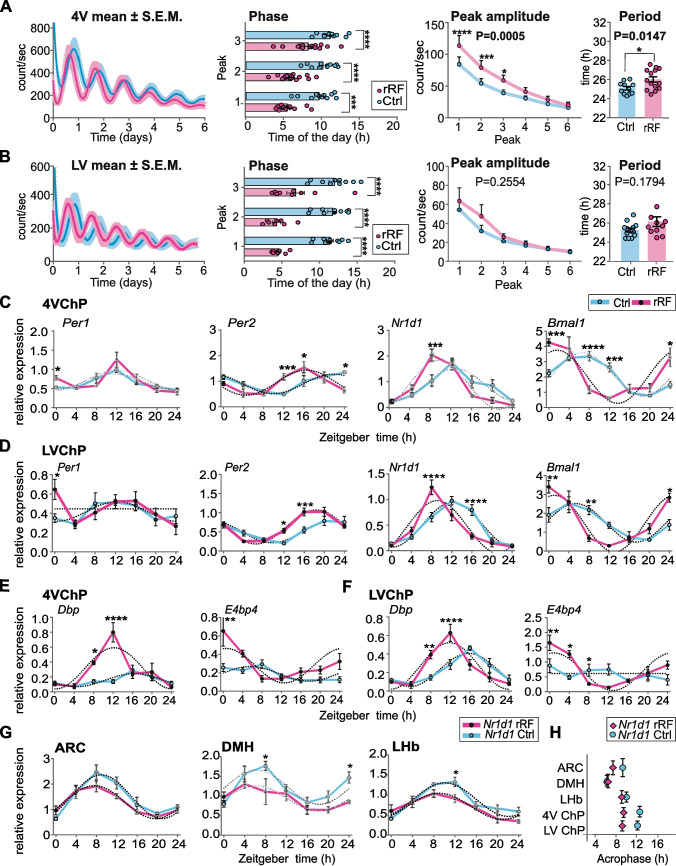
Exposure of mice to the reversed restricted feeding (rRF) affects the ChP clock. **A, B** Comparison of parameters of PER2-driven bioluminescence rhythms between the control group (Ctrl) and the rRF group for 4V ChP (**A)** and LV ChP (**B**): PER2-driven bioluminescence traces monitored over 6 days (mean ± S.E.M., *n* = 12); Phases calculated as the time, when the peak occurred (*n* = 12; 2-way ANOVA; (A) 4V ChP: *P* < 0.0001; (**B**) LV ChP: *P* < 0.0001); Amplitudes of individual peaks (*n* = 12; 2-way ANOVA; (**A**) 4V ChP: *P* = 0.0005; (**B**) LV ChP: *P* = 0.2554); Periods (*n* = 12; Mann–Whitney test, (**A**) 4V ChP: *P* = 0.0274; (**B**) LV ChP: *P* = 0.1803). **C, D** Comparison of daily clock gene expression profiles (*Per1*, *Per2*, *Nr1d1*, *and Bmal1*) between the Ctrl and the rRF groups for 4V ChP (**C**) and LV ChP (**D**). **E, F** Comparison of daily clock-controlled gene expression profiles (*Dbp*, *E4bp4*) in the Ctrl and rRF groups for (**E**) 4V ChP and (**F**) LV ChP. **G** Daily profiles of *Nr1d1* gene expression in the Ctrl and rRF groups in the arcuate nucleus (ARC), dorsomedial hypothalamus (DMH) and lateral habenula (LHb). All tissues for daily expression profiles (C-G) were collected every 4 h over 24 h period (*n* = 5 per 1 time point; For statistical comparison by 2-way ANOVA with Sidak´s multiple comparison, see Supplementary Tables S2, S3). **H** Comparison of acrophases of *Nr1d1* gene expression in different brain areas (ARC – arcuate nucleus, DMH – dorsomedial hypothalamus, LHb – lateral habenula, LV ChP – choroid plexus of the lateral ventricle, 4V ChP – choroid plexus of the fourth ventricle). Data are expressed as means ± S.E.M. *P < 0.05, **P < 0.01, ***P < 0.001, ****P < 0.0001

Next, we used the same ex vivo and in vivo approaches for testing sensitivity of the clocks in the 4V ChP and LV ChP to the rRF protocol. The bioluminescence rhythms of ChP explants prepared from animals fed ad libitum (Ctrl) and those exposed to rRF (*n* = 10 per each group; mean traces with S.E.M.) are shown in Fig. 2A and Fig. 2B, respectively. In the 4V ChP (Fig. 2A), exposure to rRF significantly advanced the phase of the first 3 peaks (2-way ANOVA; *P* < 0.0001), increased their amplitude (peak amplitude, 2-way ANOVA, *P* = 0.0005), and lengthened the period (Mann–Whitney test, *P* = 0.0147) of the rhythm compared to the Ctrl group. In the LV ChP (Fig. 2B), exposure to the rRF significantly advanced the rhythm (2-way ANOVA; *P* < 0.0001) compared to the Ctrl explants. However, the peak amplitude (2-way ANOVA, *P* = 0.2554) and period (Mann–Whitney test, *P* = 0.1794) were not affected. The phase advances induced by rRF were similar in both ChP tissues, with the exception of the 3rd peak, which was larger in LV than in 4V (2 way-ANOVA, *P* = 0.0071); the effect may relate with the fact that period slightly prolonged in 4V ChP. The ex vivo results indicate that exposure to rRF has a significant effect on phases of the clocks in both ChP tissues, but the effect on the amplitude and period is ventricle-specific, as only 4V ChP responded.

To analyze the rRF effect on clocks in both ChP tissues in more detail, we compared the daily expression profiles of clock genes (*Per1, Per2, Nr1d1, Bmal1*) (Fig. 2C, D) and clock-controlled genes (*Dbp, E4bp4)* (Fig. 2E, F) in the 4V and LV ChP samples from control and rRF-exposed mice. All genes were rhythmically expressed in rRF groups as assessed by cosinor analysis and 1-way ANOVA with the exception of *Per1* in the LV ChP (see Supplementary Fig. S2 for results of cosinor analysis and 1-way ANOVA). Comparison of daily expression profiles between Ctrl and rRF groups (for 2-way ANOVA, see Supplementary Fig. S2) revealed that exposure to rRF induced significant phase advances of all rhythmically expressed genes in both ChP tissues*.* In addition, rRF significantly increased the amplitude and advanced the phase of rhythmic expression of the clock-controlled gene *Dpb* in both ChP tissues, but the effect was more pronounced in 4V. The rRF regime had the same effect on rhythmic *E4bp4* expression in the 4V ChP and induced the circadian rhythmicity in LV ChP.

Altogether, the results of both ex vivo and in vivo experiments consistently show that the 4V and LV ChP clocks are highly sensitive to the feeding/fasting regime because exposure to rRF shifted their phases and upregulated downstream D-box element-controlled pathways regulated by the clock.

### ChP clock is more sensitive to rRF than clocks in other brain regions

To compare sensitivity of the ChP clock to rRF with other extra-SCN brain clocks, we laser-dissected samples of three brain regions involved in food intake, namely the dorsomedial hypothalamus (DMH), arcuate nucleus (ARC) and lateral habenula (LHb), from the same brains as the ChP and analyzed the daily profiles in expression of clock gene *Nr1d1* (Fig. 2G). We selected this gene as a marker of the clock because it typically shows the highest amplitude of the rhythmic expression in the extra-SCN brain regions compared to other clock genes [8]. In controls, *Nr1d1* expression exhibited significant circadian rhythms in ARC, DMH and LHb (Fig. 2G; see Supplementary Fig. S3 for results of cosinor analysis and 1-way ANOVA) and their phases were advanced by approximately 4 - 6 h compared to the rhythms of the both ChP tissues (Fig. 2H). In contrast to the ChP, exposure to rRF slightly dampened amplitudes of the rhythms in LHb and DMH compared to Ctrl group (results of 2-way ANOVA shown in the Supplementary Fig. S3) and had a negligible, if any, effect on the phases of *Nr1d1* expression in ARC, DMH and LHb (Fig. 2G, H).

Our data show that the clocks in both ChP ventricles respond to rRF protocol and are thus more sensitive to the changes in feeding/fasting regime that do not affect clocks in the other studied brain regions.

### Exposure to rRF affects expression of genes related to ChP function in a ventricle-specific manner

**Fig. 3:**
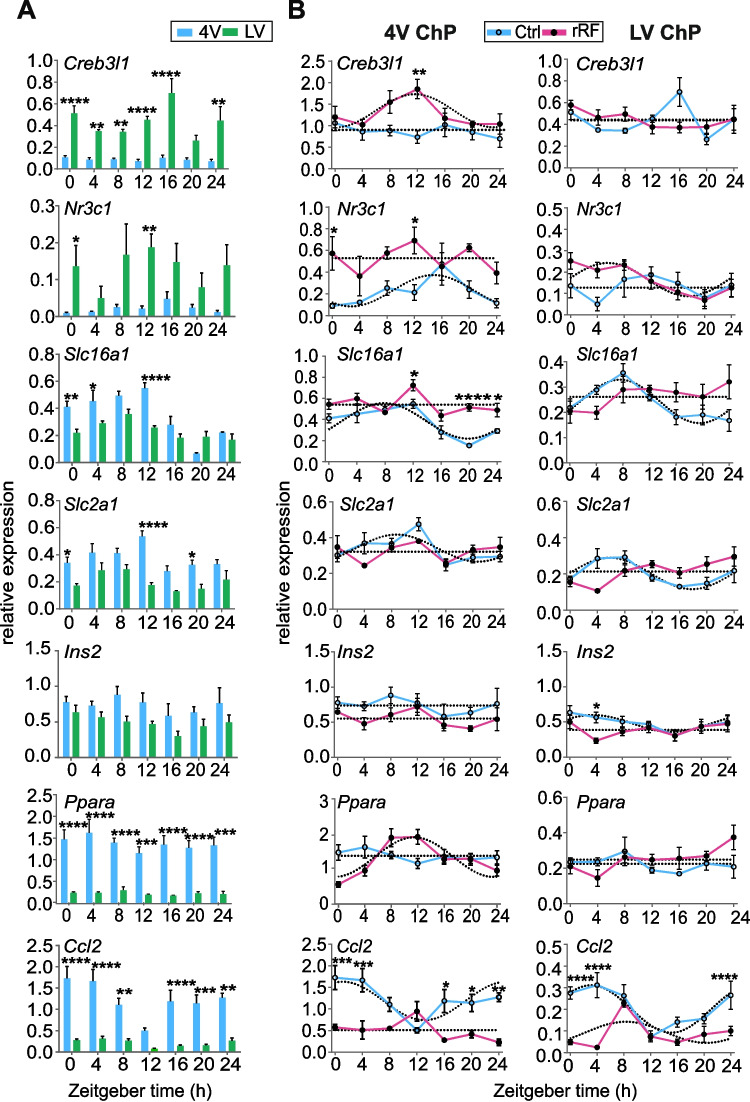
Exposure to reversed restricted feeding (rRF) affects the daily expression profiles of genes related to ChP function in a gene- and ventricle-specific manner. **A** Comparison of gene expression levels between ChP of the fourth ventricle (4V) and lateral ventricle (LV) in the control group (Ctrl). **B** Comparison of daily gene expression profiles between the Ctrl group and the rRF group. All tissues were collected every 4 h over 24 h period (*n* = 5 per 1 time point; 2-way ANOVA with Sidak´s multiple comparison; *P < 0.05, **P < 0.01, ***P < 0.001, ****P < 0.0001). Significant results of the cosinor analysis and 1-way ANOVA for the time effect were required to confirm presence of a circadian rhythm (data in Supplementary Table S4). All values are mean ± S.E.M

We assessed daily expression profiles of 10 selected genes potentially associated to the ChP function and/or sensitivity to rRF (for the selection, see Discussion), namely *Creb3l1, Nr3c1, Slc16a1, Slc2a1, Ins2, Ppara, Ccl2* (Fig. 3) *and Cldn2, Lrp1, and Il-17r* (Supplementary Fig. S1), in the 4V and LV ChP of controls and rRF-exposed animals. The results of the cosinor analysis and effect of time of the 1-way ANOVA to assess presence or absence of circadian rhythm are shown in Supplementary Fig. S4. The difference between the profiles of 4V and LV in the Ctrl group (Fig. 3A), and between the profiles of the Ctrl and rRF group for each of the ChP tissues (Fig. 3B) was assessed by the 2-way ANOVA (results are shown in Supplementary Fig. S4).

Comparison of expression levels of these genes in the 4V and LV explants from Ctrl group revealed significant ventricle-specific differences. Notably, *Creb3l1* and *Nr3c1* (Fig. 3A) were expressed with significantly higher levels in the LV ChP than the 4V ChP, whereas all other studied genes (*Slc16a1, Slc2a1, Ins2*, *Ppara, Ccl2* in Fig. 3A, and *Cldn2* and *Lrp1* in Supplementary Fig. S1) were expressed with higher levels in the 4V than the LV ChP. The *Il-17r* expression did not significantly differ between ChP of both ventricles (Supplementary Fig. S1).

Under control conditions, most of these genes were expressed non-rhythmically, because their expression profiles had (1) non-significant cosine fit and 1-way ANOVA for the effect of time (*Ppara, Cldn2, Il-17r* in both ChP tissues, and *Creb3l1* and *Lrp1* in 4 V), (2) significant cosine fit but non-significant effect of time of 1-way ANOVA (*Nr3c1* in the 4V, *Ins2* in the LV), or (3) non-significant cosine fit despite effect of time by 1-way ANOVA (*Lrp1* and *Creb3l1* in LV). Altogether, under the control conditions, the circadian rhythms were confirmed only for *Slc16a1*, *Slc2a1* and *Ccl2* in both ChP tissues (Supplementary Fig. S4).

Importantly, exposure to rRF affected expression profiles of almost all studied genes and the effect was gene-dependent (Fig. 3B). In the 4V ChP, rRF either induced rhythms in arrhythmic genes (*Cldn2*, *Lrp1*, *Ppara*), or abolished the rhythm of previously rhythmically expressed genes (*Slc16a1*, *Slc2a1, Ccl2*). Additionally, *Ccl2* was significantly downregulated. In addition, expression of *Creb3l1* and *Ins2* was significantly elevated or slightly downregulated, respectively, and the effect on other genes was only negligible, if any. In the LV ChP, the effect of rRF was much less prominent compared to 4V ChP; it induced a rhythm in expression of *Nr3c1*, abolished rhythmic expression of *Slc16a1* and *Slc2a1* and suppressed the rhythmic expression of *Ccl2*. In addition, rRF had a minor effect on the constitutive expression levels of *Lrp1* and *Ins2*.

Altogether, these results show that under control conditions, regulation of the selected function-related genes in the ChP is ventricle-specific, and that rRF exhibits ventricle- and gene-specific responses. The shifted feeding regime had larger effect on the clock in the 4V ChP. In both ChP tissues, it robustly downregulated expression of gene encoding a chemokine involved in immunoregulatory processes (*Ccl2*), and abolished rhythms of genes encoding glucose transporter (*Slc2a1*) and proton-linked monocarboxylate transporter (*Slc16a1*).

### ChP clock responds significantly to insulin

**Fig. 4:**
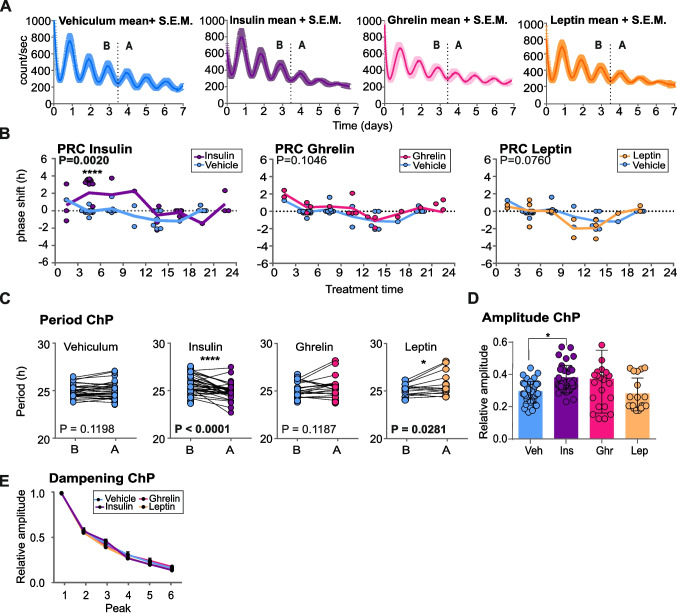
Responses of the ChP clock to insulin, leptin and ghrelin. **A** Representative PER2-driven bioluminescence traces of ChP explants (*n* = 7; mean ± S.E.M.) before and after treatment with either vehicle (H_2_O), insulin (500 nM), ghrelin (50 ng/ml) or leptin (20 nM). The dotted lines represent the treatment time. **B** Phase response curves show the effects of insulin, ghrelin and leptin on the phases of the PER2-driven bioluminescence rhythm. Explants were treated with each hormone and the corresponding vehicle at various time points relative to the peak of the bioluminescence rhythm (treatment time 12), and the resulting shifts were plotted as a function of treatment time. For statistical comparison with the effect of the vehicle, the phase shifts were binned into 3-h intervals over a 24 h cycle. The significant effect on phase was confirmed only for insulin (2-way ANOVA, *P* = 0.0020 with Sidak´s multiple comparison; *P* < 0.0001). **C** Comparison of periods before and after treatments showed that insulin significantly shortened the period (*n* = 34; Wilcoxon test, *P* < 0.0001), ghrelin had no effect (*n* = 19, *P* = 0.1187) and leptin prolonged the period (*n* = 14; Wilcoxon test, *P* = 0.0336). **D** Comparison of the relative amplitudes (ratio before and after treatment) between the vehicle and hormone treatments showed a significant increase in amplitude only after insulin treatment (*n* = 35; 1-way ANOVA, *P* =, with Tukey´s multiple comparison; *P = 0.0198). **E** Dampening of the circadian rhythm during the 6-day recordings was not affected by any of the hormones tested (*n* = 15; 2-way ANOVA, *P* = 0.2155 with Sidak´s multiple comparison test). All values are mean ± S.E.M

Because rRF may affect levels of feeding-related hormones, we tested effect of insulin, leptin and ghrelin on the ChP clock ex vivo. To spare animals needed for collection of samples to construct the full PRCs, we pooled data from 4V and LV ChP explants together. We monitored bioluminescence for 3 cycles before and after the treatment with insulin (500 nM), ghrelin (50 ng/ml), leptin (20 nM) or vehicle (H_2_O) at different time points. The bioluminescence traces (mean ± S.E.M.) before and after the treatments are shown in Fig. 4A. The treatments were performed at different time over the bioluminescence cycle, the magnitudes of resulting phase shifts were calculated and plotted relative to the bioluminescence peak (treatment time 12) to create the full PRC. For statistical comparison between the effects of each hormone and vehicle, we binned the phase responses into 3-h intervals over 24 h cycle (Fig. 4B.). The significant effect on phase of the ChP clock was confirmed only for insulin (2-way ANOVA, *P* = 0.0020), which produced significant phase advances during the first part of the cycle compared to the effect of vehicle. Moreover, insulin significantly shortened period of the ChP clock (Fig. 4C) (Wilcoxon test; *P* < 0.0001) and increased relative amplitude of the bioluminescence rhythm (Fig. 4D) (1-way ANOVA, *P* = 0.0033). Treatments with ghrelin (2-way ANOVA, *P* = 0.1046) or leptin (2-way ANOVA, *P* = 0.0760) had no significant effects on phase of the rhythm (Fig. 4B). Leptin had only slight effect on lengthening of the period (Wilcoxon test, *P* = 0.0281) and ghrelin had no effect both on period and amplitude. None of the hormones affected rate of dampening of the circadian clock (2-way ANOVA, *P* = 0.2155) (Fig. 4E).

Our data demonstrate that the ChP clock ex vivo is sensitive to insulin, which produces time-dependently significant phase advances, increases the relative amplitude and affects the period.

### Changes in glucose levels may mediate the effect of rRF on the ChP clock

**Fig. 5:**
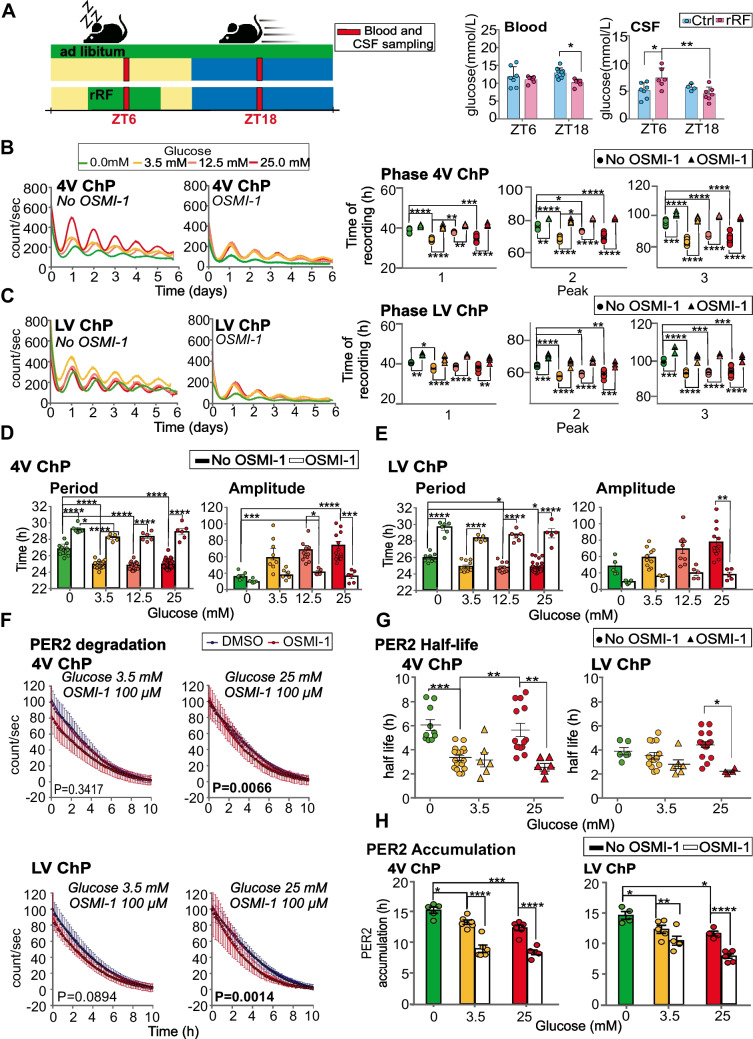
Glucose affects the ChP clock via O-GlcNAcylation. **A** Effect of rRF on glucose levels in blood and cerebrospinal fluid (*n* = 7; 2-way ANOVA with Tukey´s multiple comparison; Blood: *P = 0.0296; CSF: *P = 0.0368, **P = 0.0064). **B, C** Mean PER2-driven bioluminescence traces of 4V ChP (**B**) and LV ChP (**C**) explants cultured for 6 days in media containing 0 mM (green), 3.5 mM (yellow), 12.5 mM (pink) or 25 mM (red) glucose without OSMI-1 (left graph) or with OSMI-1 (right graph) (*n* = 7 per group). The phases of the rhythms are compared between different glucose concentrations without addition of OSMI-1 (dots in colors corresponding to glucose concentration,) and with OSMI-1 (triangles in colors corresponding to glucose concentration) (*n* = 7; 2-way ANOVA; (**B**) 4 V ChP: *P* < 0.0001, (**C**) LV ChP: *P* < 0.0001). **D, E** Comparison of periods and amplitudes for (**D**) 4 V ChP and (**E**) LV ChP explants after cultivation in glucose media without OSMI-1 (full columns) and with OSMI-1 (empty columns) (1-way ANOVA). **F** Effect of OSMI-1 (100 μM) on PER2 degradation rate of 4V and LV ChP explants cultured in media containing 3.3 mM and 25 mM glucose (F-test). DMSO was added as a control treatment. **G** Effect of glucose and OSMI-1 on the half-life of PER2. The half-life values obtained without OSMI-1 (circles) and with OSMI-1 (triangles) were compared (1-way ANOVA). **H** The effects of glucose and OSMI-1 on PER2 accumulation rate (measured as the time required for the protein to peak after CHX washout) were compared without OSMI-1 (full columns) and with OSMI-1 (open columns) (1-way ANOVA). All 1-way ANOVAs were performed with Tukey´s multiple comparison; *P < 0.05, **P < 0.01, ***P < 0.001, ****P < 0.0001. All values are means ± S.E.M

Next, we tested a hypothesis that rRF may affect the ChP clock via food intake-induced changes in glucose levels (Fig. 5). The hypothesis is based on our finding that exposure to rRF significantly affects glucose levels in the CSF (Fig. 5A) (2-way ANOVA, *P* = 0.0076) in a time-dependent matter. While in the Ctrl group the CSF glucose levels did not differ between day (ZT6) and night (ZT18) (2-way ANOVA; *P* = 0.6579), exposure to the rRF resulted in significantly higher CSF glucose levels at ZT6 than at ZT18 (2-way ANOVA; *P* = 0.0076). It was due to a significant increase of the levels at ZT6 (2-way ANOVA; *P* = 0.0392), consistent with timing of the shifted food on rRF. In the blood of the Ctrl group, glucose levels at ZT6 and ZT18 were also not different (2-way ANOVA; *P* = 0.3392), and rRF slightly decreased the glucose level at ZT18, i.e., during the fasting time when the mice had no access to food (2-way ANOVA; *P* = 0.0367).

To test whether changes in CSF glucose levels are responsible for the effect of rRF on ChP clock parameters, we cultured 4V and LV ChP explants in medium with incrementally increasing glucose concentrations (0 mM, 3.5 mM, 12.5 mM, 25 mM) and monitored bioluminescence for at least 5 days (Fig. 5B, C; data shown as “No OSMI-1”). Adding of glucose into media significantly advanced the phases of the first three peaks in both ChP tissues (2-way ANOVA; 4V ChP: *P* < 0.0001, LV ChP: *P* < 0.0001) in a dose dependent matter (Fig. 5B, C). Moreover it shortened the period (1-way ANOVA; 4V ChP: *P* < 0.0001, LV ChP: *P* = 0.0005) and increased the amplitude (1-way ANOVA, only for 4V ChP: *P* = 0.0007) of the bioluminescence rhythms (Fig. 5D, E). Our data confirm that the ChP clock ex vivo is able to respond to glucose.

### Effect of glucose on the ChP clock is mediated by changes in O-GlcNAcylation

To ascertain a possible mechanism of how glucose affects molecular clock in ChP, we tested involvement of O-GlcNAcylation (O-GlcNAc) using a specific inhibitor of O-linked N-acetylglucosaminyltransferase (OGT), OSMI-1. We compared clock parameters of bioluminescence rhythms of ChP explants cultured in media with different glucose concentrations (0 mM, 3.5 mM, 12.5 mM, 25 mM) and 100 µM OSMI-1 (Fig. 5B, C; data shown as “OSMI-1 “) with those cultured in media with glucose without OSMI-1. We found that OGT inhibition almost abolished the effects of glucose on the ChP clock. Treatment with OSMI-1 blocked the glucose-induced phase advances measured for the first three peaks of bioluminescence in the 4V ChP (Fig. 5B) and LV ChP (Fig. 5C) (2-way ANOVA; 4V ChP: *P* < 0.0001 for all three peaks, LV ChP: *P* < 0.0001 for all three peaks). OSMI-1 significantly lengthened periods of the rhythms in the 4V ChP (Fig. 5D) and LV ChP (Fig. 5E) independent of the glucose levels (2-way ANOVA; 4 V: *P* < 0.0001 for all glucose concentrations, LV: *P* < 0.0001 for all glucose concentrations). In addition, OSMI-1 also significantly reduced the amplitude of the rhythm induced by 12.5 mM and 25 mM glucose in the 4V ChP (2-way ANOVA; *P* = 0.0310 and *P* = 0.0003, respectively) (Fig. 5D) and by 25 mM glucose in the LV ChP (2-way ANOVA; *P* = 0.0035) (Fig. 5E). The effects of glucose-mediated O-GlcNAc on the ChP clock may be explained by modulation of the dynamics of PER2 turnover. We found that in presence of the highest glucose concentration (25 mM), OSMI-1 accelerated dynamics of degradation of PER2 and shortened PER2 half-life in the 4V ChP (Fig. 5F, G) as well as in the LV ChP (Fig. 5F,G) (1-way-ANOVA; 4V ChP: *P* = 0.0119, LV ChP: *P* = 0.0302). The effect was not present in 3.5 mM glucose. Accumulation of de novo translated PER2 protein was accelerated in presence of 3.5 mM and 25 mM glucose in both ChP tissues (Fig. 5H). The results are in favour of involvement of O-GlcNAc in the effect of glucose on the ChP clock.

### Changes in temperature may participate in the effect of rRF on the ChP clock

**Fig. 6:**
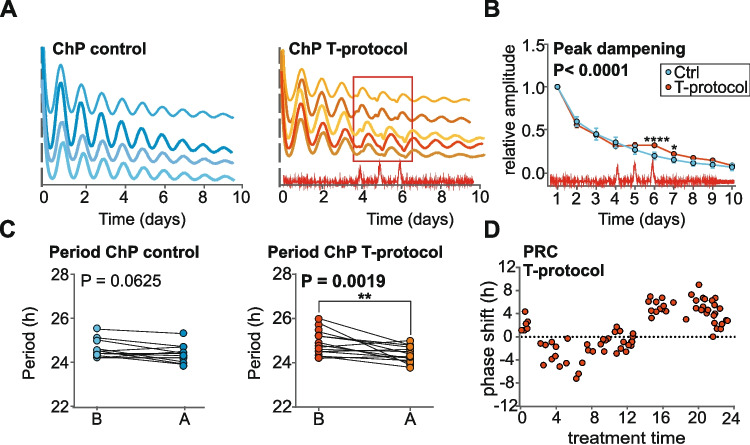
Temperature changes regulate period and phase of the ChP clock in the time dependent matter. **A** Representative PER2-driven bioluminescence traces of ChP explants cultured for 6 days in media (*n* = 5) for control group (blue) and group exposed to the T-protocol (orange – frame shows position of the temperature cycles). The red line at the x-axis shows the changes in temperature during recording. **B** Dampening of the amplitude of the circadian rhythm during the 10 days of recordings. T-protocol affected the 6th and 7th peaks (*n* = 11; 2-way ANOVA, P < 0.0001 with Sidak´s multiple comparison test; ****P < 0.0001, *P = 0.0130). All values are mean ± S.E.M. **C** Period before (**B**) and after (**A**) the T-protocol (*n* = 15; Wilcoxon test. **D** Phase response curve for the effect of T-protocol on the phase of the PER2-driven bioluminescence rhythm. ChP explants were exposed to T-protocol at various time points relative to the peak of the bioluminescence rhythm (treatment time 12), and the resulting shifts were plotted as a function of treatment time. (-) phase delays, (+) phase advances

Exposure of *mPer2*^*Luc*^ mice to rRF typically induces anticipatory activity preceding the food is provided during the inactivity time [26], which is accompanied with increase in body temperature [27, 28]. Therefore, we tested whether the clock in the ChP explants is temperature sensitive (Fig. 6). Bioluminescence was recorded in temperature conditions (T-protocol) described in Materials and Methods. The representative bioluminescence traces for control group and T-protocol exposed group are shown in Fig. 6A. The amplitude was significantly increased on the last peak of the temperature cycle and on the following peak (the 6th and 7th peak of the rhythm, respectively) compared to Ctrl group (2-way ANOVA; *P* < 0.0001) (Fig. 6B). Exposure to the T-protocol significantly shortened the period of the rhythm (Wilcoxon test; *P* = 0.0019) which was not changed in the Ctrl group (Wilcoxon test; *P* = 0.0625) (Fig. 6C). Application of the T-protocol at different phases of the individual bioluminescence cycle (treatment time 12 is the peak of bioluminescence) resulted in phase shifts, the magnitude and direction of which depended on the time of application of the T-protocol (Fig. 6D). The resulting PRC revealed that the T-protocol pulses caused phase delays when applied during the rise in PER2-bioluminescence levels and phase advances during their decline.

### Changes in osmotic equilibrium do not affect the ChP clock

In the search for other possible mechanisms that might participate in the effect of feeding on the ChP clock, we tested possible involvement of the effect of changes in osmotic equilibrium. In rodents, intake of dry chow is coupled with drinking behaviour [29], which we observed also in our *mPer2*^*Luc*^ mice via monitoring by infrared cameras to record time spent eating and drinking every hour over 24 h interval (Supplementary Fig. S2B). The monitoring confirmed that drinking of Ctrl mice fed ad libitum was aligned with the mostly nocturnal food intake. In rRF-exposed mice, the total time spent eating was shorter (Mann–Whitney test, *P* = 0.0033) and that spent drinking was slightly longer (Mann–Whitney test, *P* = 0.0268). The drinking occurred mostly during the 6-h daytime interval when food was available (ZT3-ZT9), and there was a smaller peak during the night (ZT15-ZT18), when food was not provided (2-way ANOVA, *P* = 0.0228). As rRF caused a robust burst in drinking during the daytime, we tested whether a change in osmolarity and ionic balance may affect the ChP clock (Supplementary Fig. S2C, D). We treated the ChP explants with NaCl (5.3 mM) and 0.5% sodium gluconate (SG) as a chelating agent and monitored bioluminescence for at least 3 cycles before and after the treatment. Statistical comparison did not reveal any significant effect of NaCl and SG on period of the clock in both ChP tissues (Wilcoxon test; 4 V ChP: NaCl *P* = 0.6250, SG *P* = 0.1250; LV ChP: NaCl *P* = 0.1250, SG *P* = 0.1484) (Supplementary Fig. S2C,D). In conclusion, our data do not support involvement of changes in osmolarity in the effect of rRF on the ChP clock.

## Discussion

In this study, we provide evidence that the ChP is at the forefront of brain structures whose circadian clocks respond to feeding regime. We show ventricle-specific differences in circadian parameters of 4V and LV clocks, as well as their differences in sensitivity to the rRF protocol. We investigated set of pathways related to nutritional status that may be mechanistically involved in the effect of rRF on the ChP clock. Of them, we identified rRF-induced changes in levels of insulin, glucose and body temperature associated with food-anticipatory activity as the most likely candidates for the mechanism involved.

We first compared the circadian characteristics of clocks in the 4V ChP and LV ChP, which differ in their developmental origin [30, 31] and gene expression [31]. Our results show that the clock is more robust in the 4V ChP than in the LV ChP under control conditions, as evidenced by higher amplitudes of the rhythmic in vivo profiles of the core clock gene expression (*Per1*, *Per2*, *Bmal1*, *Nr1d1*) in tissues collected over 24 h period. This does not result from a higher sensitivity of the clock to local intrinsic stimuli originating from brain and CSF, as the same difference persists ex vivo when ChP tissues are explanted from *mPer2*^*Luc*^ mice and PER2-driven bioluminescence is monitored in real time. In addition, the clock in the 4V ChP is more resilient to resetting by the explant preparation procedure than the clock in the LV ChP. The ventricle-specific difference in clock robustness may be partially explained by the constitutive upregulation of *E4bp4* in LV ChP compared to 4V ChP, as *E4bp4* encodes a repressor that binds to D-box elements localized in the promoters of *Per*, *Nr1d1* and *Ror* [32–34]*.* The repressive effect of E4BP4 on D-box elements in promoters of clock genes may override their activation by the *Dbp*-encoded activator of the D-box element, DBP, in LV ChP, affecting the clock amplitude. The other tested genes also showed ventricle-specific differences in their expression levels. Notably, *Creb3l1* and *Nr3c1* were the only genes examined that were more highly expressed in the LV ChP than in the 4V ChP and all other genes examined (*Slc16a1, Ppara, Slc2a1, Ins2*, *Ccl2*, *Cldn2* and *Lrp1*) were more highly expressed in the 4V ChP than in the LV ChP. Most genes did not pass the threshold for circadian rhythmicity, and a rhythm was confirmed only for *Slc16a1* (transport of monocarboxylates), *Slc2a1* (glucose transporter) and *Ccl2* (chemokine) in both ChP tissues under the control conditions. However, these rhythms were shallow and, despite the stringent threshold for significant rhythmicity, we cannot exclude a false positivity of the results due to sampling tissues in 4 h intervals over 24 h. In our previous circadian transcriptomic profiling study, where tissues were collected over 48 h period, none of these genes exceeded the rhythmicity threshold [11].

Exposure of mice to rRF profoundly affected the phase and the amplitude of ChP clocks and modulated expression profiles of the other examined genes in a ventricle-specific manner. The ChP explants from mice on rRF exhibited markedly phase-advanced bioluminescence rhythms compared with those from control mice fed ad libitum with shifts of approximately the same magnitudes in explants from 4V and LV. In addition, the rRF increased the amplitude and lengthened the period of the rhythm in 4V explants. We recapitulated these rRF effects in vivo, as the phases of the clock gene expression profiles (*Per2*, *Bmal1*, *Nr1d1*) were also significantly phase-advanced and their amplitudes increased in both ChP tissues; only *Per1* did not respond to rRF. Importantly, rRF increased the amplitudes and shifted the phases of the *Dbp* and *E4bp4* expression profiles in both ChP. *E4bp4* and *Dbp* are clock-controlled genes but they also provide the link for the core clock mechanism to sense changes in cellular metabolic pathways [35, 36], and their rRF-modulated levels might thus contribute to the response of the ChP clock to the rRF.

The rRF-induced phase advances of gene expression profiles in the ChP were consistent with the animals’ food-related behavior. Restricting food availability to 6 h during rest time led to a decrease in the amount of food consumed in the first (but not in the second) half of the protocol, resulting in a slight decrease in body weight (Supplementary Fig. S2A).

We have previously shown that the same protocol affected the locomotor activity of *mPer2*^*Luc*^ mice by inducing food-anticipatory behavior in expectation of food availability and their activity profiles shifted to the earlier hours (for actograms, see [26]). Numerous data demonstrate that for many (but not all) peripheral clocks throughout the body, the timing of food intake is a cue dominant over signalling from the SCN [37–39]. Here we show that the ChP clock belongs to these highly sensitive peripheral clocks. Moreover, it appears to be even more sensitive than other extra-SCN clocks in the brain, namely clocks in the DMH and ARC, i.e., regions involved in integration of metabolic signals [40–43], and the LHb, a region involved in multiple functions, including feeding behavior [44]. By using *Nr1d1* as a marker for the clock, we were able to detect circadian rhythms in these brain regions under control conditions, which was mostly not possible using other clock genes, e.g., *Per2*, that exhibit rather shallow rhythms in these brain regions. Importantly, rRF shifted *Nr1d1* expression profiles in both ChP tissues but not in DMH, ARC and LHb. However, previous studies have shown that more restrictive rRF protocols (inducing caloric restriction) trigger rhythmicity and/or shift clocks in the DMH [18, 19, 45–47] and limbic structures [48, 49]. Therefore, our relatively mild rRF protocol shifted the ChP clock, but other clocks in the brain remain insensitive.

Exposure to rRF had ventricle-specific effects on gene expression profiles in the ChP. Interestingly, expression profiles of genes that were expressed rhythmically in controls (*Ccl2*, *Slc16a1* and *Slc2a1*), were significantly affected by rRF in both ChP tissues. The expression of *Ccl2* was robustly suppressed in both tissues and became completely arrhythmic in the 4V. CCL2 is produced by ChP stromal cells [50], and its levels are suppressed during caloric restriction [51] which was slightly induced during the initial phase of the rRF protocol. However, the caloric intake was not reduced prior to collection of the ChP samples. CCL2 plays multiple roles in specific immune responses [52] and in regulating resistance to CSF drainage [53]. Therefore, by suppressing rhythmic *Ccl2* expression, rRF might affect immune state and facilitate CSF drainage to increase supply of nutrients to the brain, as an adaptation to limited access to food. The upregulation of transcription factor *Creb3l1*, which is a known regulator of cell secretory capacity [54], could significantly modulate the secretion of bioactive molecules from the 4V ChP into the CSF. The upregulation of *Nr3c1* may result from rRF-modulated glucocorticoid hormone levels in plasma, as previously documented [39, 55]. Importantly, rRF upregulated *Creb3l1* and *Nr3c1* only in the 4V ChP, where under control conditions both genes were expressed less than in the LV. The modulation of *Slc16a1* and *Slc2a1* expression profiles in both ChP tissues suggests an effect of rRF on the transport of monocarboxylates (lactic acid and ketone bodies) and glucose, respectively, across ChP epithelial cells. The slight downregulation of *Ins2* expression in both ChP tissues may reflect an adaptation of insulin production in the ChP [56] to rRF-induced insulin levels in plasma, which we previously documented [39]. Another mechanism by which feeding regime may influence *Ins2* expression in the ChP is serotonergic input from the raphe nuclei into the ChP [57]. The pathway may be modulated by changes in feeding regime [58], and it has been shown that serotonergic signals modulate insulin secretion from the ChP epithelium [56].

To ascertain the mechanisms of how rRF affects the ChP clock, we first tested the effects of insulin and glucose, two obvious players in nutrient responses to rRF, on the circadian clock in ChP using an ex vivo approach. Insulin could signal changes in feeding patterns to the ChP clock, as we have previously shown that its peak in plasma shifts according to feeding time on the rRF [39]. In addition, previous study showed a link between food-induced insulin and AKT-mTORC1-SREBP-1c pathway as a multi-tissue mechanism affecting cellular clocks [59]. Indeed, insulin induced significant time-dependent phase advances, increased the amplitude and shortened the period of the PER2-driven bioluminescence rhythm, demonstrating its ability to entrain the ChP clock. The effect of insulin on the clock may involve its modulation of expression of insulin-sensitive gene *E4bp4* [35] that was elevated in both ChP tissues due to rRF. In contrast to insulin, treatments with other nutrient-related hormones, which also bind receptors in the ChP, ghrelin [60] and leptin [61], had no significant effect on the clock.

The direct effect of glucose on peripheral clocks has previously been studied in other cell types, but with variable results. Glucose induced a down-regulation of *Per1* and *Per2* expression in rat-1 fibroblasts [62] or had no effect on the amplitude of PER2-driven bioluminescence in mouse lung fibroblasts [59]. We investigated the effect of glucose on the ChP clock because we found that the rRF significantly increased glucose levels in the CSF during food intake (at ZT6). Glucose affected the ChP clock with a larger effect in 4V than in LV. Culturing the explants in a medium with added glucose (3.5, 12.5 and 25 mM) significantly increased the amplitude, shortened the period and advanced the phases of the bioluminescence rhythms. This effect on the clock can be explained by the significant effect of glucose on shortening PER2 half-life and accelerating de novo PER2 accumulation in the ChP. Therefore, our data strongly suggest that the clock in ChP tissue has a selective sensitivity to glucose that may help in its synchronization according to feeding time. We found that at least part of the glucose effects on the ChP clock is mediated via the O-linked N-acetylglucosamine (O-GlcNAc) protein posttranslational modification, which is activated by the hexosamine biosynthetic pathway (HBP). The HBP is a glucose metabolism pathway that participates in nutrient sensing and its activation results in the synthesis of UDP-GlcNAC, a key substrate for the O-GlcNAc [63, 64]. The O-GlcNAc regulates the circadian clock via competition with clock protein phosphorylation [65] and inhibition of BMAL1/CLOCK ubiquitination [66]. In the presence of elevated glucose levels PER2 O-GlcNAcylation increases, which in turn blocks the phosphorylation and ubiquitination processes [65]. The O-GlcNAc inhibition significantly lengthened the period of the ChP clock independent of glucose levels in both ChP tissues, which may result from employment of O-GlcNAc modification of BMAL1 [66]. We show that selective pharmacological inhibition of O-GlcNAc transferase (OGT) by OSMI-1 [67] blocks the effect of glucose on increasing the amplitude and advancing the clock in the ChP; these effects were more pronounced in the 4V than in the LV ChP, similarly to the effect of glucose. Altogether, our data show that glucose may affect amplitude and phase of the ChP clock (both parameters affected by rRF) at least partially by modification of clock protein PER2 via activation of O-GlcNAc pathway. The partial effect of OGT inhibition is reasonable considering that equilibrium between protein glycosylation, phosphorylation and ubiquitination is crucial for adjusting the pace of the circadian clock [68–70]. Nutrient-related glucose-induced disruption in this equilibrium can affect the balance between phosphorylation targeting clock proteins for degradation and O-GlcNAcylation modifying them for their stabilisation [70].

Apart from nutrient signals, we have also investigated other possible mechanisms potentially involved in the effect of rRF on the ChP clock. We have previously shown that rRF induces food-anticipatory activity in *mPer2*^*Luc*^ mice [26], which is accompanied by changes in body temperature [27]. Temperature changes have been shown to synchronize various clocks in vitro [71–75]. We found that the clock in ChP explants is highly sensitive to temperature changes in the physiological range. Exposure to three 24-h cycles with 3-h pulses of elevated temperature from 37 °C to 39 °C (T-protocol) increased the amplitude and shortened the period of the ChP clock. In addition, the T-protocol induced phase shifts of the ChP clock, the magnitude and direction of which were dependent on the timing of T-protocol application. The significant effects on both period and phase confirm the ability of body temperature, which increases with the food anticipatory activity, to entrain the ChP clock.

Altogether, our data suggest that feeding behavior related increase in body temperature may participate on the adaptation of the ChP clock to changes in feeding regime. On the other hand, we did not see effect of changes in osmolarity and ionic balance that accompany the huge rRF-induced redistribution of drinking pattern, on the ChP clock.

Considering the important role of the circadian clock in the temporal regulation of ChP physiological functions [6, 11, 22, 76], it is important to determine its sensitivity and responsiveness to lifestyle related stimuli that challenge both the internal synchrony of the circadian system and metabolism. Our results provide strong evidence that the ChP clock is highly sensitive to changes in feeding patterns via sensitivity to a complex of stimuli that include temporal changes in insulin and glucose levels and behavioral activity elevated body temperature.

## Supplementary Information

Below is the link to the electronic supplementary material.

Supplementary file1 (DOCX 14 KB)

Supplementary file2 (DOCX 19 KB)

Supplementary file3 (DOCX 15 KB)

Supplementary file4 (DOCX 22 KB)

Supplementary file5 Effect of reversed restricted feeding (rRF) on the daily expression profiles of *Cldn2*, *Lrp1*, *and Il-17*. **A **Comparison of expression levels between ChP of the fourth ventricle (4V) and lateral ventricle (LV) in the control group (Ctrl). **B** Comparison of daily gene expression profiles between the Ctrl group and the rRF group. All tissues were collected every 4 h over 24 h period (*n* = 5 per 1 time point; 2-way ANOVA with Sidak´s multiple comparison; data in Supplementary Table S4; *P < 0.05, **P < 0.01, ***P<0.001, ****P < 0.0001). Significant results of the cosinor analysis and 1-way ANOVA for the time effect were required to confirm presence of a circadian rhythm (data in Supplementary Table S4). All values are mean ± S.E.M (PDF 1425 KB)

Supplementary file6 rRF-modulated feeding and drinking behavior and effect of ionic balance change on the ChP clock.** A** Effect of rRF on the amount of food consumed (2-way ANOVA; *n* =12; *P*= 0.0047, with Sidak´s multiple comparison test (*P < 0.05, **P < 0.01,***P <0.001, ****P < 0.0001) and change in body weight (paired t-test; *n* = 18; P (Ctrl) = 0.0673, *P* (rRF) = 0.004) before (**B**) and after (**A**) the protocol. **B **rRF-modulated feeding pattern (2-way ANOVA; *P* =0.0107) is closely related to changes in drinking pattern (2-way ANOVA; *P* = 0.0228; Sidak´s multiple comparison; *P < 0.05,**P < 0.01, ***P <0.001, ****P < 0.0001). The total amounts of drinking (Mann-Whitney test; *P = 0.0268) and feeding (Mann-Whitney test; **P = 0.0033) times are affected by rRF. **C **and** D** Changes in period (*n* = 6; Wilcoxon test) and relative amplitude (*n* = 5; 1-way ANOVA with Tukey´s multiple comparison) in 4 V ChP (**C**) and LV ChP (**D**) after treatment with vehicle, NaCl and sodium gluconate. All values are mean ± S.E.M (PDF 1471 KB)

## Data Availability

All data are presented.

## References

[1] MacAulay N, Keep RF, Zeuthen T (2022) Cerebrospinal fluid production by the choroid plexus: a century of barrier research revisited. Fluids Barriers CNS 19(1):26 35317823 10.1186/s12987-022-00323-1 PMC8941821

[2] Marques F et al (2017) The choroid plexus in health and in disease: dialogues into and out of the brain. Neurobiol Dis 107:32–40 27546055 10.1016/j.nbd.2016.08.011

[3] Segal MB (1993) Extracellular and cerebrospinal fluids. J Inherit Metab Dis 16(4):617–638 8412010 10.1007/BF00711896

[4] Ghersi-Egea JF et al (2018) Molecular anatomy and functions of the choroidal blood-cerebrospinal fluid barrier in health and disease. Acta Neuropathol 135(3):337–361 29368213 10.1007/s00401-018-1807-1

[5] Quintela T et al (2018) The choroid plexus harbors a circadian oscillator modulated by estrogens. Chronobiol Int 35(2):270–279 29172740 10.1080/07420528.2017.1400978

[6] Myung J et al (2018) The choroid plexus is an important circadian clock component. Nat Commun 9(1):1062 29540683 10.1038/s41467-018-03507-2 PMC5852131

[7] Yamaguchi T et al (2020) Characterization of the circadian oscillator in the choroid plexus of rats. Biochem Biophys Res Commun 524(2):497–501 32008747 10.1016/j.bbrc.2020.01.125

[8] Liska K et al (2021) Glucocorticoids reset circadian clock in choroid plexus via period genes. J Endocrinol 248(2):155–166 33350982 10.1530/JOE-20-0526

[9] Takahashi JS et al (2008) The genetics of mammalian circadian order and disorder: implications for physiology and disease. Nat Rev Genet 9(10):764–775 18802415 10.1038/nrg2430 PMC3758473

[10] Schibler U, Naef F (2005) Cellular oscillators: rhythmic gene expression and metabolism. Curr Opin Cell Biol 17(2):223–229 15780601 10.1016/j.ceb.2005.01.007

[11] Sladek M et al (2024) The circadian clock in the choroid plexus drives rhythms in multiple cellular processes under the control of the suprachiasmatic nucleus. Fluids Barriers Cns 21(1):46 38802875 10.1186/s12987-024-00547-3 PMC11131265

[12] Damiola F et al (2000) Restricted feeding uncouples circadian oscillators in peripheral tissues from the central pacemaker in the suprachiasmatic nucleus. Genes Dev 14(23):2950–2961 11114885 10.1101/gad.183500 PMC317100

[13] Feillet CA et al (2008) Forebrain oscillators ticking with different clock hands. Mol Cell Neurosci 37(2):209–221 17996461 10.1016/j.mcn.2007.09.010

[14] Abe M et al (2002) Circadian rhythms in isolated brain regions. J Neurosci 22(1):350–356 11756518 10.1523/JNEUROSCI.22-01-00350.2002 PMC6757616

[15] Orozco-Solis R et al (2016) The circadian clock in the ventromedial hypothalamus controls cyclic energy expenditure. Cell Metab 23(3):467–478 26959185 10.1016/j.cmet.2016.02.003 PMC5373494

[16] Wise RA (2004) Dopamine, learning and motivation. Nat Rev Neurosci 5(6):483–494 15152198 10.1038/nrn1406

[17] Berridge KC (2007) The debate over dopamine’s role in reward: the case for incentive salience. Psychopharmacology 191(3):391–431 17072591 10.1007/s00213-006-0578-x

[18] Verwey M et al (2007) Differential regulation of the expression of period2 protein in the limbic forebrain and dorsomedial hypothalamus by daily limited access to highly palatable food in food-deprived and free-fed rats. Neuroscience 147(2):277–285 17544223 10.1016/j.neuroscience.2007.04.044

[19] Verwey M et al (2008) Region-specific modulation of PER2 expression in the limbic forebrain and hypothalamus by nighttime restricted feeding in rats. Neurosci Lett 440(1):54–58 18541376 10.1016/j.neulet.2008.05.043

[20] Verwey M, Lam GYM, Amir S (2009) Circadian rhythms of PERIOD1 expression in the dorsomedial hypothalamic nucleus in the absence of entrained food-anticipatory activity rhythms in rats. Eur J Neurosci 29(11):2217–2222 19490091 10.1111/j.1460-9568.2009.06766.x

[21] Challet E (2019) The circadian regulation of food intake. Nat Rev Endocrinol 15(7):393–405 31073218 10.1038/s41574-019-0210-x

[22] Fame RM et al (2023) Defining diurnal fluctuations in mouse choroid plexus and CSF at high molecular, spatial, and temporal resolution. Nat Commun 14(1):3720 37349305 10.1038/s41467-023-39326-3 PMC10287727

[23] Houdek P, Sumova A (2014) In vivo initiation of clock gene expression rhythmicity in fetal rat suprachiasmatic nuclei. PLoS One 9(9):e107360 25255311 10.1371/journal.pone.0107360 PMC4177808

[24] Sladek M et al (2012) Early chronotype and tissue-specific alterations of circadian clock function in spontaneously hypertensive rats. PLoS One 7(10):e46951 23056539 10.1371/journal.pone.0046951 PMC3462770

[25] Noguchi T et al (2020) Circadian rhythm bifurcation induces flexible phase resetting by reducing circadian amplitude. Eur J Neurosci 51(12):2329–2342 30044021 10.1111/ejn.14086

[26] Ralph MR et al (2021) Targeted modification of the Per2 clock gene alters circadian function in mPer2luciferase (mPer2Luc) mice. PLoS Comput Biol 17(5):e1008987 34048425 10.1371/journal.pcbi.1008987 PMC8191895

[27] Szentirmai E et al (2010) Restricted feeding-induced sleep, activity, and body temperature changes in normal and preproghrelin-deficient mice. Am J Physiol Regul Integr Comp Physiol 298(2):R467–R477 19939974 10.1152/ajpregu.00557.2009 PMC2828180

[28] Chavan R et al (2016) Liver-derived ketone bodies are necessary for food anticipation. Nat Commun 7:10580 26838474 10.1038/ncomms10580 PMC4742855

[29] Kraly FS (1984) Physiology of drinking elicited by eating. Psychol Rev 91(4):478–490 6390479

[30] Netsky MG, Shuangshoti S (2013) The choroid plexus in health and disease. Elsevier Science, Butterworth-Heinemann

[31] Dani N et al (2021) A cellular and spatial map of the choroid plexus across brain ventricles and ages. Cell 184(11):3056–3074 (**e21**) 33932339 10.1016/j.cell.2021.04.003 PMC8214809

[32] Mitsui S et al (2001) Antagonistic role of E4BP4 and PAR proteins in the circadian oscillatory mechanism. Genes Dev 15(8):995–1006 11316793 10.1101/gad.873501 PMC312673

[33] Ohno T, Onishi Y, Ishida N (2007) A novel E4BP4 element drives circadian expression of mPeriod2. Nucleic Acids Res 35(2):648–655 17182630 10.1093/nar/gkl868 PMC1802629

[34] Sato TK et al (2006) Feedback repression is required for mammalian circadian clock function. Nat Genet 38(3):312–319 16474406 10.1038/ng1745 PMC1994933

[35] Tong X et al (2010) Transcriptional repressor E4-binding protein 4 (E4BP4) regulates metabolic hormone fibroblast growth factor 21 (FGF21) during circadian cycles and feeding. J Biol Chem 285(47):36401–36409 20851878 10.1074/jbc.M110.172866 PMC2978569

[36] Yoshitane H et al (2019) Functional D-box sequences reset the circadian clock and drive mRNA rhythms. Commun Biol 2(1):300 31428688 10.1038/s42003-019-0522-3 PMC6687812

[37] Sladek M et al (2007) Insight into the circadian clock within rat colonic epithelial cells. Gastroenterology 133(4):1240–1249 17675004 10.1053/j.gastro.2007.05.053

[38] Polidarova L et al (2013) Increased sensitivity of the circadian system to temporal changes in the feeding regime of spontaneously hypertensive rats - a potential role for Bmal2 in the liver. PLoS One 8(9):e75690 24086613 10.1371/journal.pone.0075690 PMC3783415

[39] Honzlová P et al (2022) Misaligned feeding schedule elicits divergent circadian reorganizations in endo- and exocrine pancreas clocks. Cell Mol Life Sci 79(6):318 35622158 10.1007/s00018-022-04354-7 PMC11072313

[40] Sainsbury A, Cooney GJ, Herzog H (2002) Hypothalamic regulation of energy homeostasis. Best Pract Res Clin Endocrinol Metab 16(4):623–637 12468411 10.1053/beem.2002.0230

[41] Timper K, Bruning JC (2017) Hypothalamic circuits regulating appetite and energy homeostasis: pathways to obesity. Dis Model Mech 10(6):679–689 28592656 10.1242/dmm.026609 PMC5483000

[42] Zhang Y et al (2011) Leptin-receptor-expressing neurons in the dorsomedial hypothalamus and median preoptic area regulate sympathetic brown adipose tissue circuits. J Neurosci 31(5):1873–1884 21289197 10.1523/JNEUROSCI.3223-10.2011 PMC3069639

[43] Imoto D et al (2021) Refeeding activates neurons in the dorsomedial hypothalamus to inhibit food intake and promote positive valence. Mol Metab 54:101366 34728342 10.1016/j.molmet.2021.101366 PMC8609163

[44] Ip CK et al (2023) Critical role of lateral habenula circuits in the control of stress-induced palatable food consumption. Neuron 111(16):2583–2600 (**e6**) 37295418 10.1016/j.neuron.2023.05.010

[45] Mieda M et al (2006) The dorsomedial hypothalamic nucleus as a putative food-entrainable circadian pacemaker. Proc Natl Acad Sci U S A 103(32):12150–12155 16880388 10.1073/pnas.0604189103 PMC1567710

[46] Fuller PM, Lu J, Saper CB (2008) Differential rescue of light- and food-entrainable circadian rhythms. Science 320(5879):1074–1077 18497298 10.1126/science.1153277 PMC3489954

[47] Moriya T et al (2009) The dorsomedial hypothalamic nucleus is not necessary for food-anticipatory circadian rhythms of behavior, temperature or clock gene expression in mice. Eur J Neurosci 29(7):1447–1460 19519629 10.1111/j.1460-9568.2009.06697.x

[48] Angeles-Castellanos M, Mendoza J, Escobar C (2007) Restricted feeding schedules phase shift daily rhythms of c-Fos and protein Per1 immunoreactivity in corticolimbic regions in rats. Neuroscience 144(1):344–355 17045749 10.1016/j.neuroscience.2006.08.064

[49] Lamont EW et al (2007) Restricted access to food, but not sucrose, saccharine, or salt, synchronizes the expression of period2 protein in the limbic forebrain. Neuroscience 144(2):402–411 17067744 10.1016/j.neuroscience.2006.09.027

[50] Shimada A, Hasegawa-Ishii S (2021) Increased cytokine expression in the choroid plexus stroma and epithelium in response to endotoxin-induced systemic inflammation in mice. Toxicol Rep 8:520–528 33747797 10.1016/j.toxrep.2021.03.002 PMC7973137

[51] Jordan S et al (2019) Dietary intake regulates the circulating inflammatory monocyte pool. Cell 178(5):1102–1114 (**e17**) 31442403 10.1016/j.cell.2019.07.050 PMC7357241

[52] Lin Z et al (2022) CCL2: an important cytokine in normal and pathological pregnancies: a review. Front Immunol 13:1053457 36685497 10.3389/fimmu.2022.1053457 PMC9852914

[53] Alimajstorovic Z et al (2020) Cerebrospinal fluid dynamics modulation by diet and cytokines in rats. Fluids Barriers Cns 17(1):10 10.1186/s12987-020-0168-z PMC7008525 32036786

[54] Chan CP, Kok KH, Jin DY (2011) CREB3 subfamily transcription factors are not created equal: Recent insights from global analyses and animal models. Cell Biosci 1(1):6 10.1186/2045-3701-1-6 PMC3116243 21711675

[55] Van Cauter E et al (1991) Modulation of glucose regulation and insulin secretion by circadian rhythmicity and sleep. J Clin Invest 88(3):934–942 1885778 10.1172/JCI115396 PMC295490

[56] Mazucanti CH et al (2019) Release of insulin produced by the choroid plexis is regulated by serotonergic signaling. JCI Insight 4(23):e131682 31647782 10.1172/jci.insight.131682 PMC6962018

[57] Moskowitz MA et al (1979) Raphe origin of serotonin-containing neurons within choroid-plexus of the rat. Brain Res 169(3):590–594 156057 10.1016/0006-8993(79)90410-4

[58] Saeed R et al (2022) Behavioral, hormonal, and serotonergic responses to different restricted feeding schedules in rats. Int J Tryptophan Res 15:11786469221104728 35757086 10.1177/11786469221104729 PMC9218908

[59] Crosby P et al (2019) Insulin/IGF-1 drives PERIOD synthesis to entrain circadian rhythms with feeding time. Cell 177(4):896–909 (**e20**) 31030999 10.1016/j.cell.2019.02.017 PMC6506277

[60] Uriarte M et al (2021) Circulating ghrelin crosses the blood-cerebrospinal fluid barrier via growth hormone secretagogue receptor dependent and independent mechanisms. Mol Cell Endocrinol 538:111449 34478806 10.1016/j.mce.2021.111449

[61] Di Spiezio A et al (2018) The LepR-mediated leptin transport across brain barriers controls food reward. Mol Metab 8:13–22 29254602 10.1016/j.molmet.2017.12.001 PMC5985039

[62] Hirota T et al (2002) Glucose down-regulates Per1 and Per2 mRNA levels and induces circadian gene expression in cultured Rat-1 fibroblasts. J Biol Chem 277(46):44244–44251 12213820 10.1074/jbc.M206233200

[63] Lam C et al (2021) The hexosamine biosynthetic pathway and cancer: current knowledge and future therapeutic strategies. Cancer Lett 503:11–18 33484754 10.1016/j.canlet.2021.01.010

[64] Cork GK, Thompson J, Slawson C (2018) Real talk: the inter-play between the mTOR, AMPK, and hexosamine biosynthetic pathways in cell signaling. Front Endocrinol (Lausanne) 9:522 30237786 10.3389/fendo.2018.00522 PMC6136272

[65] Kaasik K et al (2013) Glucose sensor O-GlcNAcylation coordinates with phosphorylation to regulate circadian clock. Cell Metab 17(2):291–302 23395175 10.1016/j.cmet.2012.12.017 PMC3597447

[66] Li MD et al (2013) O-GlcNAc signaling entrains the circadian clock by inhibiting BMAL1/CLOCK ubiquitination. Cell Metab 17(2):303–310 23395176 10.1016/j.cmet.2012.12.015 PMC3647362

[67] Ortiz-Meoz RF et al (2015) A small molecule that inhibits OGT activity in cells. ACS Chem Biol 10(6):1392–1397 25751766 10.1021/acschembio.5b00004 PMC4475500

[68] Li YH et al (2019) O-GlcNAcylation of PERIOD regulates its interaction with CLOCK and timing of circadian transcriptional repression. PLoS Genet 15(1):e1007953 30703153 10.1371/journal.pgen.1007953 PMC6372208

[69] Wang Y et al (2020) Nuclear localized O-fucosyltransferase SPY facilitates PRR5 proteolysis to fine-tune the pace of arabidopsis circadian clock. Mol Plant 13(3):446–458 31899321 10.1016/j.molp.2019.12.013 PMC7058189

[70] Hirano A, Fu YH, Ptácek LJ (2016) The intricate dance of post-translational modifications in the rhythm of life. Nat Struct Mol Biol 23(12):1053–1060 27922612 10.1038/nsmb.3326

[71] Moraes MN et al (2017) Melanopsin, a canonical light receptor, mediates thermal activation of clock genes. Sci Rep 7(1):13977 29070825 10.1038/s41598-017-13939-3 PMC5656685

[72] Tamaru T et al (2011) Synchronization of circadian Per2 rhythms and HSF1-BMAL1: CLOCK interaction in mouse fibroblasts after short-term heat shock pulse. PLoS One 6(9):e24521 21915348 10.1371/journal.pone.0024521 PMC3168500

[73] Buhr ED, Yoo SH, Takahashi JS (2010) Temperature as a universal resetting cue for mammalian circadian oscillators. Science 330(6002):379–385 20947768 10.1126/science.1195262 PMC3625727

[74] Brown SA et al (2002) Rhythms of mammalian body temperature can sustain peripheral circadian clocks. Curr Biol 12(18):1574–1583 12372249 10.1016/s0960-9822(02)01145-4

[75] Sladek M, Sumova A (2013) Entrainment of spontaneously hypertensive rat fibroblasts by temperature cycles. PLoS One 8(10):e77010 24116198 10.1371/journal.pone.0077010 PMC3792033

[76] Christensen J, Li C, Mychasiuk R (2022) Choroid plexus function in neurological homeostasis and disorders: the awakening of the circadian clocks and orexins. J Cereb Blood Flow Metab 42(7):1163–1175 35296175 10.1177/0271678X221082786 PMC9207490

