# Supplementary material for: Feeding regime synchronizes circadian clock in choroid plexus - insight into a complex mechanism
Source: Cell Mol Life Sci. 2025 Jun 23;82(1):247. doi: 10.1007/s00018-025-05798-3 (PMC12185859; doi:10.1007/s00018-025-05798-3)
Supplement: Supplementary file 1 — Supplementary file1 (DOCX 14 KB) [file 18_2025_5798_MOESM1_ESM.docx]

**Supplementary Table S1** *List of forward (F) and reverse (R) primers used for gene expression analysis by RT qPCR*

| *B2m* |  | *F:TCTCACTGACCGGCCTGTATGCTATC* |
| --- | --- | --- |
|  |  | *R:* *AATGTGAGGCGGGTGGAACTGTG* |
| *Tbp* | | *F: GTTCTTAGACTTCAAGATCCAG* |
|  |  | *R: TTCTGGGTTTGATCATTCTG* |
| *Pgk1* | | *F: CTATCATAGGTGGTGGAGAC* |
|  |  | *R: ACACTAGGTTGACTTAGGAG* |
| *Per1* | | *F: GTTCTCATAGTTCCTCTTCTG* |
|  |  | *R: GTGAGTTTGTACTCTTGCTG* |
| *Per2* | | *F: CTTTCACTGTAAGAAGGACG* |
|  |  | *R:* *CTGAGTGAAAGAATCTAAGCC* |
| *Bmal1* | | *F: GGTTCTCACCAAGAATAGAAG* |
|  |  | *R: TCTATTCTTGGTGAGAACCC* |
| *Nr1d1* | | *F: AACATTACCAAGCTGAATGG* |
|  |  | *R: CTGGATATTCTGTTGGATGC* |
| *Dbp* | | *F: TCAACCAATCATGAAGAAGG* |
|  |  | *R: TGCTTCATTGTTCTTGTACC* |
| *E4bp4* | | *F:* *GCCCTTTCTTTTCCCCCTCA* |
|  |  | *R:* *TGTCCGGCACAGGGTAAATC* |
| *Ins2* | | *F:* *AGCAGGAAGGTTATTGTTTC* |
|  |  | *R:* *ACATGGGTGTGTAGAAGAAG* |
| *Cldn2* | | *F:* *GGTTTCATTCCCATCTGTAAG* |
|  |  | *R:* *AAGAAGGCATCTAGAAAACG* |
| *Lrp1* | | *F:* *CCTACCTAGACTACATCGAG* |
|  |  | *R: GCGTAGAGATAGTTCTCAAAC* |
| *Slc16a1* | | *F:* *CATTGGTGTTATTGGAGGTC* |
|  |  | *R:* *GAAAGCCTGATTAAGTGGAG* |
| *Slc2a1* | | *F:* *AAGTCCAGGAGGATATTCAG* |
|  |  | *R:* *CTACAGTGTGGAGATAGGAG* |
| *Ppara* | | *F:* *GATGTCACACAATGCAATTC* |
|  |  | *R: CAGTTTCCGAATCTTTCAGG* |
| *Creb3I1* | | *F:* *TAAGAACAAGATTTCTGCCC* |
|  |  | *R: TCTCTGATGTATATGTCTCCAC* |
| *Nr3c1* | | *F:* *GAAAACCTTACTGCTTCTCTC* |
|  |  | *R:* *GATTTTCAACCACATCATGC* |
| *Il-17r* | | *F:* *CAAATACCACAGTTCCCAAG* |
|  |  | *R:* *GAGTCATCACCATGTTTCTC* |
| *Ccl2* | | *F:* *CAAGATGATCCCAATGAGTAG* |
|  |  | *R:* *TTGGTGACAAAAACTACAGC* |
