# Supplementary material for: Feeding regime synchronizes circadian clock in choroid plexus - insight into a complex mechanism
Source: Cell Mol Life Sci. 2025 Jun 23;82(1):247. doi: 10.1007/s00018-025-05798-3 (PMC12185859; doi:10.1007/s00018-025-05798-3)
Supplement: Supplementary file 2 — Supplementary file2 (DOCX 19 KB) [file 18_2025_5798_MOESM2_ESM.docx]

**Supplementary Table S2** *Cosinor analysis, 2-way ANOVA and 1-way ANOVA of the clock gene expression profiles in controls (ad libitum) and rRF-exposed mice in ChP.*

|  | *Per1* | *Per2* | *Nr1d1* | *Bmal1* | *Dbp* | *E4bp4* |
| --- | --- | --- | --- | --- | --- | --- |
| *AD LIBITUM 4V* | |  |  |  |  |  |
| acro ± SE | 11.41 ± 0.75 | 21.85 ± 0.69 | 12.59 ± 0.61 | 6.71 ± 0.31 | 16.39 ± 0.91 | 5.63 ± 0.95 |
| amp ± SE | 0.2097 ± 0.0381 | 0.3823 ± 0.0665 | 0.6487 ± 0.0955 | 1.4220 ± 0.1253 | 0.0876 ± 0.0220 | 0.0800 ± 0.0216 |
| R2 | 0.5022 | 0.5251 | 0.606 | 0.8111 | 0.3456 | 0.3136 |
| P | **< 0.0001** | **< 0.0001** | **< 0.0001** | **< 0.0001** | **0.0,017** | **0.0035** |
| \| 1-way ANOVA \| **0.0001** \| **0.0008** \| **< 0.0001** \| **< 0.0001** \| 0.5048 \| **0.0055** \| \| --- \| --- \| --- \| --- \| --- \| --- \| --- \| | **0.0001** | **0.0008** | **< 0.0001** | **< 0.0001** | **0.0151** | **0.0055** |
| *AD LIBITUM LV* | |  |  |  |  |  |
| acro ± SE | 12.30 ± 1.38 | 21.69 ± 0.61 | 12.17 ± 0.36 | 5.37 ± 0.51 | 15.76 ± 0.49 | - |
| amp ± SE | 0.1012 ± 0.0329 | 0.2826 ± 0.0436 | 0.4375 ± 0.0370 | 1.0230 ± 0.1643 | 0.1790 ± 0.0235 |  |
| R2 | 0.2338 | 0.5776 | 0.8185 | 0.5814 | 0.6597 |  |
| P | 0.0161 | **< 0.0001** | **< 0.0001** | **< 0.0001** | **< 0.0001** | 0.5671 |
| 1-way ANOVA | 0.3977 | **0.0002** | **< 0.0001** | **0.0003** | **< 0.0001** | 0.3771 |
| *rRF 4V* |  |  |  |  |  |  |
| acro ± SE | 12.11 ± 1.19 | 16.48 ± 0.62 | 9.41 ± 0.38 | 1.63 ± 0.58 | 12.18 ± 0.73 | 0.62 ± 1.01 |
| amp ± SE | 0.2599 ± 0.0716 | 0.4888 ± 0.0838 | 0.961 ± 0.0931 | 1.738 ± 0.2475 | 0.2760 ± 0.0477 | 0.1868 ± 0.0445 |
| R2 | 0.3195 | 0.5342 | 0.7762 | 0.6155 | 0.5187 | 0.3626 |
| P | **0.0046** | **< 0.0001** | **< 0.0001** | **< 0.0001** | **< 0.0001** | **0.0009** |
| 1-way ANOVA | **0.0003** | **0.0002** | **< 0.0001** | **< 0.0001** | **< 0.0001** | **0.0004** |
| 2-way ANOVA |  |  |  |  |  |  |
| Interaction | **0.0380** | **0.0001** | **< 0.0001** | **< 0.0001** | **< 0.0001** | **0.0011** |
| Time | **< 0.0001** | **< 0.0001** | **< 0.0001** | **< 0.0001** | **< 0.0001** | **< 0.0001** |
| Group | 0.2259 | 0.6137 | 0.8742 | 0.4426 | **0.0004** | **0.0009** |
| *rRF LV* |  |  |  |  |  |  |
| acro ± SE | - | 18.41 ± 0.30 | 9.12 ± 0.44 | 1.10 ± 0.48 | 11.87 ± 0.53 | 0.86 ± 0.61 |
| amp ± SE |  | 0.4420 ± 0.0397 | 0.5081 ± 0.0586 | 1.502 ± 0.1722 | 0.2505 ± 0.0327 | 0.6344 ± 0.0897 |
| R2 |  | 0.8052 | 0.7107 | 0.7113 | 0.6681 | 0.6251 |
| P | 0.4349 | **< 0.0001** | **< 0.0001** | **< 0.0001** | **< 0.0001** | **< 0.0001** |
| 1-way ANOVA | 0.0325 | **< 0.0001** | **< 0.0001** | **< 0.0001** | **< 0.0001** | **< 0.0001** |
| 2-way ANOVA |  |  |  |  |  |  |
| Interaction | 0.1458 | **0.0004** | **< 0.0001** | **< 0.0001** | **< 0.0001** | **< 0.0001** |
| Time | **0.0078** | **< 0.0001** | **< 0.0001** | **< 0.0001** | **< 0.0001** | **< 0.0001** |
| Group | 0.4378 | **0.0242** | 0.4145 | 0.4145 | 0.2911 | 0.0953 |

Acrophases (acro) in hours and amplitudes (amp) are shown for daily profiles with significant cosinor fits as assessed by R^2^ and significance level (P) in control (*ad libitum*) and experimental (restricted feeding; rRF) animals in fourth ventricle (4V) and lateral ventricle (LV) choroid plexus (ChP). Presence of circadian rhythm in daily profiles of gene expression was assessed also by 1-way ANOVA for the effect of time. Differences analysed by 2-way ANOVA between control (*ad libitum*) and experimental (restricted feeding; rRF) profiles were compared by factors of interaction, time and group. Results are shown as p values.
