# Supplementary material for: Feeding regime synchronizes circadian clock in choroid plexus - insight into a complex mechanism
Source: Cell Mol Life Sci. 2025 Jun 23;82(1):247. doi: 10.1007/s00018-025-05798-3 (PMC12185859; doi:10.1007/s00018-025-05798-3)
Supplement: Supplementary file 3 — Supplementary file3 (DOCX 15 KB) [file 18_2025_5798_MOESM3_ESM.docx]

**Supplementary Table S3** *Cosinor analysis, 2-way ANOVA and 1-way ANOVA of the Nr1d1 expression profiles in selected brain structures*

|  | *DMH* | *LHb* | *ARC* |
| --- | --- | --- | --- |
| *AD LIBITUM* |  |  |  |
| acro ± SE | 6.24 ± 0.73 | 10.07 ± 0.47 | 8.95 ± 0.52 |
| amp ± SE | ­0.4392 ± 0.0946 | ­0.4185 ± 0.0480 | ­0.8599 ± 0.1174 |
| R^2^ | 0.4025 | 0.7059 | 0.6363 |
| P | **0.0003** | **< 0.0001** | **< 0.0001** |
| 1-way ANOVA | **0.0001** | **< 0.0001** | **< 0.0001** |
| *rRF* |  |  |  |
| acro ± SE | 6.28 ± 0.95 | 9.00 ± 0.66 | 7.62 ± 0.43 |
| amp ± SE | ­0.3245 ± 0.0912 | ­0.3255 ± 0.0564 | ­0.6481 ± 0.0771 |
| R^2^ | 0.2833 | 0.5141 | 0.6961 |
| P | 0.0048 | **< 0.0001** | **< 0.0001** |
| 1-way ANOVA | 0.0724 | **0.0004** | **< 0.0001** |
| 2-way ANOVA |  |  |  |
| Interaction | 0.1116 | 0.0518 | 0.2230 |
| Time | **< 0.0001** | **< 0.0001** | **< 0.0001** |
| Group | **0.0012** | **0.0050** | 0.0885 |

Acrophases (acro) in hours and amplitudes (amp) are shown for daily profiles with significant cosinor fits as assessed by R^2^ and significance level (P) in control (*ad libitum*) and experimental (restricted feeding; rRF) animals in Dorsomedial hypothalamus (*DMH*), Lateral Habenula (*LHb*) and Nucleus arcuatus (*ARC).* Presence of circadian rhythm in daily profiles of gene expression was assessed also by 1-way ANOVA for the effect of time. Differences analysed by 2-way ANOVA between control (*ad libitum*) and experimental (restricted feeding; rRF) profiles were compared by factors of interaction, time and group. Results are shown as p values.
