# Supplementary material for: Feeding regime synchronizes circadian clock in choroid plexus - insight into a complex mechanism
Source: Cell Mol Life Sci. 2025 Jun 23;82(1):247. doi: 10.1007/s00018-025-05798-3 (PMC12185859; doi:10.1007/s00018-025-05798-3)
Supplement: Supplementary file 4 — Supplementary file4 (DOCX 22 KB) [file 18_2025_5798_MOESM4_ESM.docx]

**Supplementary Table S4.** *Cosinor analysis, 2-way ANOVA and 1-way ANOVA of the selected ChP function-related gene expression profiles in controls (Ad libitum) and rRF-exposed mice.*

|  | *Ins2* | *Cldn2* | *Lrp1* | *Slc16a1* | *Slc2a1* |
| --- | --- | --- | --- | --- | --- |
| *AD LIBITUM 4V* |  |  |  |  |  |
| acro ± SE | - | - | - | 8.365 ± 0.6379 | ­8.75 ± 1.173 |
| amp ± SE |  |  |  | ­0.2128 ± 0.0365 | ­0.0854 ± 0.0265 |
| R^2^ |  |  |  | 0.5325 | 0.2583 |
| P | 0.247 | 0.1176 | 0.1925 | **< 0.0001** | **0.0113** |
| 1-way ANOVA | 0.6575 | 0.2462 | 0.2590 | **< 0.0001** | **0.0076** |
| *AD LIBITUM LV* |  |  |  |  |  |
| acro ± SE | ­3.531 ± 2.71 | - | - | 7.55 ± 0.71 | ­5.85 ± 0.79 |
| amp ± SE | 0.0599 ± 0.0429 |  |  | ­0.0860 ± 0.0171 | 0.0866 ± 0.0198 |
| R^2^ | 0.1988 |  |  | 0.4511 | 0.3812 |
| P | 0.036 | 0.4501 | 0.8819 | **< 0.0001** | **0.0006** |
| 1-way ANOVA | 0.1697 | 0.8545 | 0.0068 | **0.0015** | **0.0151** |
| *rRF 4V* |  |  |  |  |  |
| acro ± SE | - | ­11.17 ± 0.98 | ­11.76 ± 1.57 | - | - |
| amp ± SE |  | ­0.1695 ± 0.0396 | ­0.1092 ± 0.0403 |  |  |
| R^2^ |  | 0.3723 | 0.1914 |  |  |
| P | 0.3551 | **0.0007** | **0.0371** | 0.3745 | 0.9779 |
| 1-way ANOVA | 0.2268 | **0.0004** | **0.0059** | 0.0033 | 0.1237 |
| 2-way ANOVA |  |  |  |  |  |
| Interaction | 0.9341 | 0.1752 | 0.0829 | **0.0138** | 0.1428 |
| Time | 0.1325 | **< 0.0001** | **0.0092** | **< 0.0001** | **0.0024** |
| Group | **0.0023** | 0.0777 | 0.1116 | **< 0.0001** | 0.5329 |
|  |  |  |  |  |  |
| *rRF LV* |  |  |  |  |  |
| acro ± SE | - | - | - | - | - |
| amp ± SE |  |  |  |  |  |
| R^2^ |  |  |  |  |  |
| P | 0.3839 | 0.6069 | 0.2584 | 0.4754 | 0.2306 |
| 1-way ANOVA | 0.1769 | 0.6386 | 0.7623 | 0.4257 | 0.0078 |
| 2-way ANOVA |  |  |  |  |  |
| Interaction | 0.3281 | 0.6970 | 0.9460 | **0.0273** | **0.0008** |
| Time | 0.0664 | 0.8330 | 0.7184 | 0.0807 | 0.0423 |
| Group | **0.0251** | 0.3117 | **0.0071** | 0.2008 | 0.6112 |

|  | *Ppara* | *Creb3l1* | *Nr3c1* | *Ccl2* | *Il-17r* |
| --- | --- | --- | --- | --- | --- |
| *AD LIBITUM 4V* |  |  |  |  |  |
| acro ± SE | - | - | 14.78 ± 1.28 | 1.111 ± 1.09 | - |
| amp ± SE |  |  | ­0.0144 ±0.0048 | 0.4440 ± 0.1185 |  |
| R^2^ |  |  | 0.2549 | 0.3197 |  |
| P | 0.3612 | 0.8912 | 0.0218 | **0.0031** | 0.4755 |
| 1-way ANOVA | 0.7688 | 0.8324 | 0.2836 | **0.0091** | 0.4081 |
| *AD LIBITUM LV* |  |  |  |  |  |
| acro ± SE | - | - | - | 3.117 ± 0.76 | - |
| amp ± SE |  |  |  | 0.1096 ± 0.0219 |  |
| R^2^ |  |  |  | 0.4489 |  |
| P | 0.2966 | 0.2092 | 0.3246 | **< 0.0001** | 0.6419 |
| 1-way ANOVA | 0.5469 | 0.0043 | 0.4352 | **0.0018** | 0.1510 |
| *rRF 4V* |  |  |  |  |  |
| acro ± SE | ­11.46 ± 0.72 | ­13.23 ± 1.36 | - | - | 9.31 ± 1.40 |
| amp ± SE | ­0.5635 ± 0.0959 | ­0.0334 ± 0.0110 |  |  | 0.1820 ± 0.0657 |
| R^2^ | 0.5269 | 0.2316 |  |  | 0.200 |
| P | **< 0.0001** | 0.0168 | 0.4990 | 0.0752 | 0.0314 |
| 1-way ANOVA | **< 0.0001** | 0.0640 | 0.4524 | 0.0323 | 0.0761 |
| 2-way ANOVA |  |  |  |  |  |
| Interaction | **0.0002** | 0.1253 | 0.3064 | **0.0007** | **0.0437** |
| Time | **0.0251** | 0.3360 | 0.2574 | 0.1152 | 0.5980 |
| Group | 0.2957 | **0.0006** | **< 0.0001** | **< 0.0001** | 0.7487 |
| *rRF LV* |  |  |  |  |  |
| acro ± SE | ­- | - | 5.58 ± 0.97 | 8.32 ± 1.40 | - |
| amp ± SE |  |  | 0.0778 ± 0.0219 | 0.0488 ± 0.0182 |  |
| R^2^ |  |  | 0.2883 | 0.2008 |  |
| P | 0.5088 | 0.0845 | **0.0051** | **0.0387** | 0.0727 |
| 1-way ANOVA | 0.0800 | 0.2179 | **0.0086** | **< 0.0001** | 0.2276 |
| 2-way ANOVA |  |  |  |  |  |
| Interaction | 0.1270 | **0.0099** | 0.1318 | **0.0004** | 0.6013 |
| Time | 0.2624 | **0.0191** | 0.0783 | **< 0.0001** | **0.0386** |
| Group | 0.2241 | 0.8546 | 0.1425 | **< 0.0001** | 0.0904 |

Acrophases (acro) in hours and amplitudes (amp) are shown for daily profiles with significant cosinor fits as assessed by R^2^ and significance level (P) in control (*ad libitum*) and experimental (restricted feeding; rRF) animals in fourth ventricle (4V) and lateral ventricle (LV) choroid plexus (ChP). Presence of circadian rhythm in daily profiles of gene expression was assessed also by 1-way ANOVA for the effect of time. Differences analysed by 2-way ANOVA between control (*ad libitum*) and experimental (restricted feeding; rRF) profiles were compared by factors of interaction, time and group. Results are shown as p values.
