# Supplementary figures and images for: Feeding regime synchronizes circadian clock in choroid plexus - insight into a complex mechanism
Source: Cell Mol Life Sci. 2025 Jun 23;82(1):247. doi: 10.1007/s00018-025-05798-3 (PMC12185859; doi:10.1007/s00018-025-05798-3)

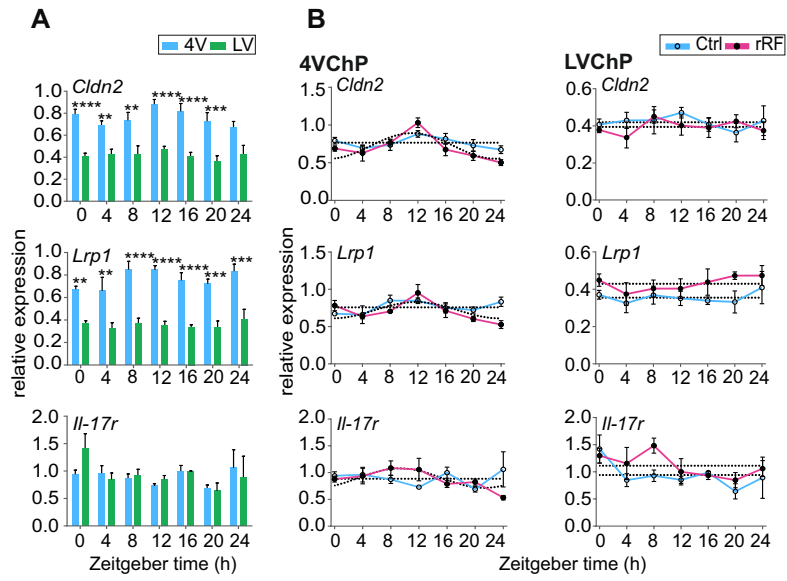

Supplement: Supplementary file 5 — Supplementary file5 Effect of reversed restricted feeding (rRF) on the daily expression profiles of Cldn2, Lrp1, and Il-17. A Comparison of expression levels between ChP of the fourth ventricle (4V) and lateral ventricle (LV) in the control group (Ctrl). B Comparison of daily gene expression profiles between the Ctrl group and the rRF group. All tissues were collected every 4 h over 24 h period (n = 5 per 1 time point; 2-way ANOVA with Sidak´s multiple comparison; data in Supplementary Table S4; *P < 0.05, **P < 0.01, ***P<0.001, ****P < 0.0001). Significant results of the cosinor analysis and 1-way ANOVA for the time effect were required to confirm presence of a circadian rhythm (data in Supplementary Table S4). All values are mean ± S.E.M (PDF 1425 KB) [file 18_2025_5798_MOESM5_ESM.pdf]

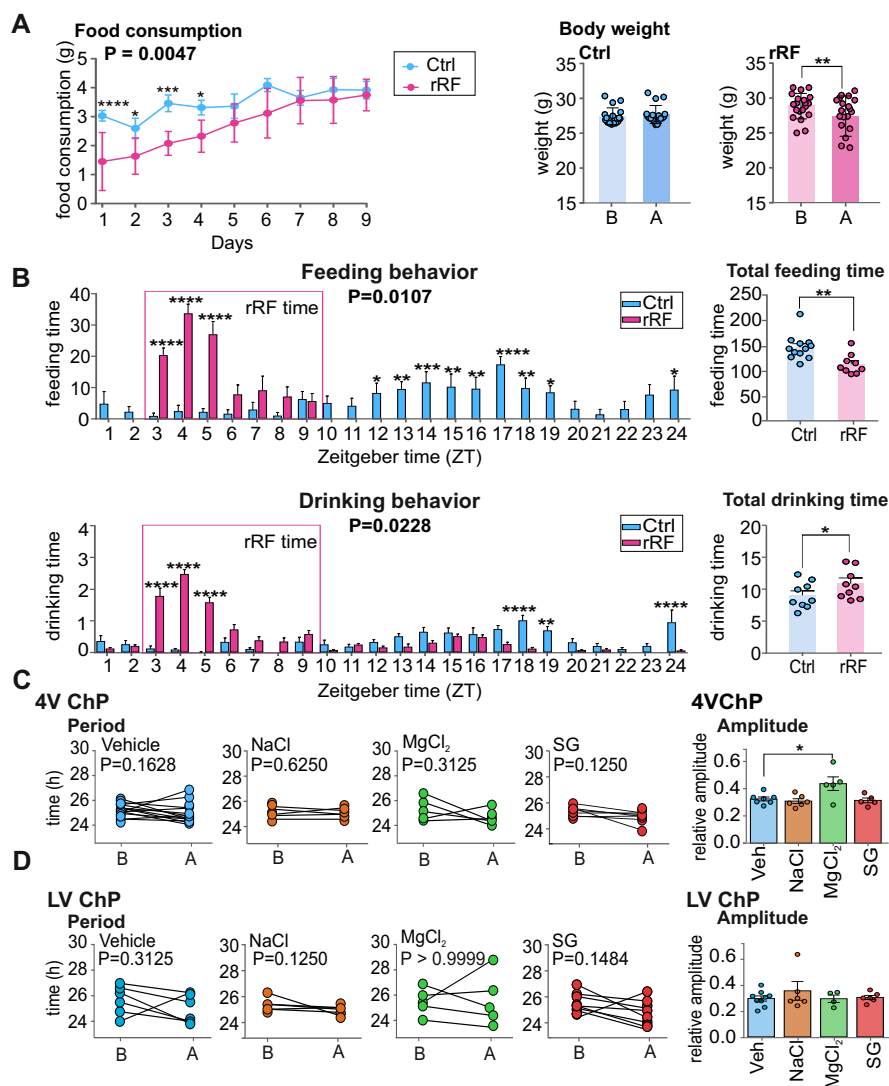

Supplement: Supplementary file 6 — Supplementary file6 rRF-modulated feeding and drinking behavior and effect of ionic balance change on the ChP clock. A Effect of rRF on the amount of food consumed (2-way ANOVA; n =12; P= 0.0047, with Sidak´s multiple comparison test (*P < 0.05, **P < 0.01,***P <0.001, ****P < 0.0001) and change in body weight (paired t-test; n = 18; P (Ctrl) = 0.0673, P (rRF) = 0.004) before (B) and after (A) the protocol. B rRF-modulated feeding pattern (2-way ANOVA; P =0.0107) is closely related to changes in drinking pattern (2-way ANOVA; P = 0.0228; Sidak´s multiple comparison; *P < 0.05,**P < 0.01, ***P <0.001, ****P < 0.0001). The total amounts of drinking (Mann-Whitney test; *P = 0.0268) and feeding (Mann-Whitney test; **P = 0.0033) times are affected by rRF. C and D Changes in period (n = 6; Wilcoxon test) and relative amplitude (n = 5; 1-way ANOVA with Tukey´s multiple comparison) in 4 V ChP (C) and LV ChP (D) after treatment with vehicle, NaCl and sodium gluconate. All values are mean ± S.E.M (PDF 1471 KB) [file 18_2025_5798_MOESM6_ESM.pdf]
